# Author Correction: Genetic effects on the timing of parturition and links to fetal birth weight

**DOI:** 10.1038/s41588-023-01412-z

**Published:** 2023-05-10

**Authors:** Pol Solé-Navais, Christopher Flatley, Valgerdur Steinthorsdottir, Marc Vaudel, Julius Juodakis, Jing Chen, Triin Laisk, Abigail L. LaBella, David Westergaard, Jonas Bacelis, Ben Brumpton, Line Skotte, Maria C. Borges, Øyvind Helgeland, Anubha Mahajan, Matthias Wielscher, Frederick Lin, Catherine Briggs, Carol A. Wang, Gunn-Helen Moen, Robin N. Beaumont, Jonathan P. Bradfield, Abin Abraham, Gudmar Thorleifsson, Maiken E. Gabrielsen, Sisse R. Ostrowski, Dominika Modzelewska, Ellen A. Nohr, Elina Hypponen, Amit Srivastava, Octavious Talbot, Catherine Allard, Scott M. Williams, Ramkumar Menon, Beverley M. Shields, Gardar Sveinbjornsson, Huan Xu, Mads Melbye, William Lowe, Luigi Bouchard, Emily Oken, Ole B. Pedersen, Daniel F. Gudbjartsson, Christian Erikstrup, Erik Sørensen, Early Growth Genetics Consortium, Mark I. McCarthy, Estonian Biobank Research Team, Danish Blood Donor Study Genomic Consortium, Rolv T. Lie, Kari Teramo, Mikko Hallman, Thorhildur Juliusdottir, Hakon Hakonarson, Henrik Ullum, Andrew T. Hattersley, Line Sletner, Mario Merialdi, Sheryl L. Rifas-Shiman, Thora Steingrimsdottir, Denise Scholtens, Christine Power, Jane West, Mette Nyegaard, John A. Capra, Anne H. Skogholt, Per Magnus, Ole A. Andreassen, Unnur Thorsteinsdottir, Struan F. A. Grant, Elisabeth Qvigstad, Craig E. Pennell, Marie-France Hivert, Geoffrey M. Hayes, Marjo-Riitta Jarvelin, Mark I. McCarthy, Deborah A. Lawlor, Henriette S. Nielsen, Reedik Mägi, Antonis Rokas, Kristian Hveem, Kari Stefansson, Bjarke Feenstra, Pål Njolstad, Louis J. Muglia, Rachel M. Freathy, Stefan Johansson, Ge Zhang, Bo Jacobsson

**Affiliations:** 1 Department of Obstetrics and Gynaecology, Sahlgrenska Academy, Institute of Clinical Science, University of Gothenburg, Gothenburg, Sweden; 2 deCODE genetics/Amgen, Reykjavik, Iceland; 3 Center for Diabetes Research, Department of Clinical Science, University of Bergen, Bergen, Norway; 4 Division of Biomedical Informatics, Cincinnati Children’s Hospital Medical Center, Cincinnati, OH USA; 5 Department of Pediatrics, University of Cincinnati College of Medicine, Cincinnati, OH USA; 6 Estonian Genome Centre, Institute of Genomics, University of Tartu, Tartu, Estonia; 7 Department of Biological Sciences, Vanderbilt University, Nashville, TN USA; 8 Novo Nordisk Foundation Center for Protein Research, University of Copenhagen, Copenhagen, Denmark; 9 Department of Obstetrics and Gynaecology, Copenhagen University Hospital Hvidovre, Hvidovre, Denmark; 10 Methods and Analysis, Statistics Denmark, Copenhagen, Denmark; 11 K.G. Jebsen Center for Genetic Epidemiology, Department of Public Health and Nursing, Norwegian University of Science and Technology, Trondheim, Norway; 12 Department of Epidemiology Research, Statens Serum Institut, Copenhagen, Denmark; 13 MRC Integrative Epidemiology Unit, University of Bristol, Bristol, UK; 14 Population Health Sciences, Bristol Medical School, University of Bristol, Bristol, UK; 15 Department of Genetics and Bioinformatics, Health Data and Digitalization, Norwegian Institute of Public Health, Oslo, Norway; 16 Wellcome Trust Centre for Human Genetics, University of Oxford, Oxford, UK; 17 Department of Epidemiology and Biostatistics, MRC-PHE Centre for Environment and Health, School of Public Health, Imperial College London, London, UK; 18 Department of Dermatology, Medical University of Vienna, Vienna, Austria; 19 Northwestern University Feinberg School of Medicine, Chicago, IL USA; 20 Department of Population Medicine, Harvard Medical School, Harvard Pilgrim Health Care Institute, Boston, MA USA; 21 School of Medicine and Public Health, College of Health, Medicine and Wellbeing, The University of Newcastle, Callaghan, New South Wales Australia; 22 Hunter Medical Research Institute, New Lambton Heights, New South Wales Australia; 23 Institute of Clinical Medicine, Faculty of Medicine, University of Oslo, Oslo, Norway; 24 University of Queensland Diamantina Institute, University of Queensland, Woolloongabba, Australia; 25 Institute of Biomedical and Clinical Science, College of Medicine and Health, University of Exeter, Exeter, UK; 26 Quantinuum Research LLC, Wayne, PA USA; 27 Children’s Hospital of Philadelphia, Philadelphia, PA USA; 28 Department of Clinical Immunology, Copenhagen University Hospital - Rigshospitalet, Copenhagen, Denmark; 29 Department of Clinical Medicine, University of Copenhagen, Copenhagen, Denmark; 30 Research Unit of Gynecology and Obstetrics, Institute of Clinical Research, University of Southern Denmark, Odense, Denmark; 31 Australian Centre for Precision Health, Uni Clinical & Health Sciences, University of South Australia, Adelaide, Australia; 32 South Australian Health and Medical Research Institute, Adelaide, Australia; 33 Division of Human Genetics, Center for the Prevention of Preterm Birth, Perinatal Institute, Cincinnati Children’s Hospital Medical Center, Cincinnati, OH USA; 34 Centre de recherche du Centre hospitalier universitaire de Sherbrooke (CHUS), Sherbrooke, Québec Canada; 35 Department of Population and Quantitative Health Sciences, School of Medicine, Case Western Reserve University, Cleveland, OH USA; 36 Department of Obstetrics and Gynaecology, University of Texas Medical Branch, Galveston, TX USA; 37 Centre for Fertility and Health, Norwegian Institute of Public Health, Oslo, Norway; 38 Department of Genetics, Stanford University School of Medicine, Stanford, CA USA; 39 Department of Biochemistry and Functional Genomics, Faculty of Medicine and Health Sciences, Université de Sherbrooke, Sherbrooke, Québec, Canada; 40 Clinical Department of Laboratory Medicine, Centre intégré universitaire de santé et de services sociaux (CIUSSS) du Saguenay-Lac-St-Jean - Hôpital Universitaire de Chicoutimi, Saguenay, Québec Canada; 41 Department of Clinical Immunology, Zealand University Hospital, Køge, Denmark; 42 School of Engineering and Natural Sciences, University of Iceland, Reykjavik, Iceland; 43 Department of Clinical Immunology, Aarhus University Hospital, Aarhus, Denmark; 44 Department of Clinical Medicine, Faculty of Health, University of Aarhus, Aarhus, Denmark; 45 Department of Global Public Health and Primary Care, University of Bergen, Bergen, Norway; 46 Department of Obstetrics and Gynecology, Helsinki University Central Hospital, Helsinki, Finland; 47 University of Helsinki, Helsinki, Finland; 48 PEDEGO Research Unit and Medical Research Center Oulu, University of Oulu, Oulu, Finland; 49 Department of Children and Adolescents, Oulu University Hospital, Oulu, Finland; 50 The Center for Applied Genomics, Children’s Hospital of Philadelphia, Philadelphia, PA USA; 51 Department of Pediatrics, The Perelman School of Medicine, University of Pennsylvania, Philadelphia, PA USA; 52 Division of Human Genetics, Children’s Hospital of Philadelphia, Philadelphia, PA USA; 53 Division of Pulmonary Medicine, Children’s Hospital of Philadelphia, Philadelphia, PA USA; 54 State Serum Institute, Copenhagen, Denmark; 55 Department of Pediatric and Adolescents Medicine, Akershus University Hospital, Lørenskog, Norway; 56 Maternal Newborn Health Innovations, PBC, Geneva, Switzerland; 57 Faculty of Medicine, University of Iceland, Reykjavik, Iceland; 58 Department of Obstetrics and Gynecology, Landspitali – The National University Hospital of Iceland, Reykjavik, Iceland; 59 Population, Policy, Practice. Great Ormond Street Institute of Child Health, University College London, London, UK; 60 Bradford Institute for Health Research, Bradford Teaching Hospitals NHS Foundation Trust, Bradford, UK; 61 Department of Health Science and Technology, Aalborg University, Aalborg, Denmark; 62 Bakar Computational Health Sciences Institute and Department of Epidemiology and Statistics, University of California San Francisco, San Francisco, CA USA; 63 NORMENT Centre, University of Oslo, Oslo, Norway; 64 Division of Mental Health and Addiction, Oslo University Hospital, Oslo, Norway; 65 Center for Spatial and Functional Genomics Children’s Hospital of Philadelphia, Philadelphia, PA USA; 66 Divisions of Human Genetics and Endocrinology and Diabetes, Children’s Hospital of Philadelphia, Philadelphia, PA USA; 67 Department of Genetics, The Perelman School of Medicine, University of Pennsylvania, Philadelphia, PA USA; 68 Department of Endocrinology, Morbid Obesity and Preventive Medicine, Oslo University Hospital, Oslo, Norway; 69 Diabetes Unit, Massachusetts General Hospital, Boston, MA USA; 70 Center for Life Course Health Research, Faculty of Medicine, University of Oulu, Oulu, Finland; 71 Biocenter of Oulu, University of Oulu, Linnanmaa, Oulu Finland; 72 Department of Life Sciences, College of Health and Life Sciences, Brunel University London, Uxbridge, UK; 73 NIHR Bristol Biomedical Research Centre, Bristol, UK; 74 The Recurrent Pregnancy Loss Unit, The Capital Region, Copenhagen University Hospitals Rigshospitalet & Hvidovre Hospital, Hvidovre, Denmark; 75 Department of Biomedical Informatics, Vanderbilt University School of Medicine, Nashville, TN USA; 76 Vanderbilt Genetics Institute, Vanderbilt University, Nashville, TN USA; 77 HUNT Research Centre, Department of Public Health and Nursing, Norwegian University of Science and Technology, Levanger, Norway; 78 Department of Medicine, Levanger Hospital, Nord-Trøndelag Hospital Trust, Levanger, Norway; 79 Children and Youth Clinic, Haukeland University Hospital, Bergen, Norway; 80 Department of Medical Genetics, Haukeland University Hospital, Bergen, Norway; 81 Present Address: Genentech, South San Francisco, CA USA

**Keywords:** Genetics research, Genome-wide association studies

Correction to: *Nature Genetics* 10.1038/s41588-023-01343-9. Published online 3 April 2023.

In the version of this article originally published, the surname of author Stefan Johansson was misspelled as Johanson. In the abstract and “Genome-wide association analyses” section of the Results, the number of loci associated with preterm birth was shown as six instead of seven. In addition, there was a production error in the Figure 1a *y*-axis units shown below 0, where “10” and “20” were shown as negative numbers. The errors have been corrected in the HTML and PDF versions of the article.

