# Supplementary material for: Genetic effects on the timing of parturition and links to fetal birth weight
Source: Nat Genet. 2023 Apr 3;55(4):559–67. doi: 10.1038/s41588-023-01343-9 (PMC10101852; doi:10.1038/s41588-023-01343-9)
Supplement: Supplementary file 1 — Supplementary Note and Supplementary Figures 1–16. [file 41588_2023_1343_MOESM1_ESM.pdf]

nature genetics

Article

https://doi.org/10.1038/s41588-023-01343-9

Genetic effects on the timing of parturition and links to fetal birth weight

In the format provided by the authors and unedited

Supplementary Note

Supplementary Methods
- Quality control of individual GWAS summary statistics
- Enrichment analysis
- Resolving effect origin
- Colocalization
- Female reproductive traits
- Locus pleiotropy at 3q21
- Gestational duration and preterm delivery polygenic scores
- Latent causal variable analysis
- Multi-trait conditional analysis
- Mendelian randomization
- Evolutionary analysis
- Software

Consortium author lists and affiliations
- Danish Blood Donor Study Genomic Consortium
- Estonian Biobank Research Team
- Early Growth Genetics

Description of participating cohorts
- 23andMe
- Avon Longitudinal Study of Parents and Children
- Born in Bradford
- British 1958 Birth Cohort (1958BC-T1DGC and 1958BC-WTCCC2)
- Children's Hospital of Philadelphia (CHOP)
- Danish Blood Donor Study
- deCODE
- Danish National Birth Cohort - DNBC
- Estonian Genome Center of the University of Tartu
- Exeter Family of Childhood Health (EFSOCH)
- FIN
- GPN
- HAPO
- HUNT
- Northern Finland Birth Cohort 1966
- Project Viva
- STORK
- STORK Groruddalen
- The Genetics of Glucose regulation in Gestation and Growth (Gen3G)
- The Norwegian Mother, Father and Child Cohort Study

The Preterm birth Genome Project

Cohort acknowledgements
23andMe
ALSPAC
BiB
CHOP
DBDS
DNBC
EFSOCH
EGCUT
FIN
FINNGEN
Gen3G
GPN
HAPO
HUNT
MoBa
NFBC
PGP
Project Viva
STORK-G
STORK
UK-Biobank
WTCCC58BC

Bibliography

Supplementary figures

Supplementary Methods

Quality control of individual GWAS summary statistics

Summary statistics for each individual cohort were stored centrally and underwent quality control procedures before meta-analysis. Genetic variant ids were converted to 'CHR:POS:REF:EFF' (positions were mapped to the Genome Reference Consortium Human Build 37, hg19), where EFF was the alphabetically higher allele - effect sizes were aligned accordingly. Alleles for insertion/deletions were coded as 'I/D', respectively. Only sequence variants from the Haplotype Reference Consortium panel or 1000 Genomes Project were included in the meta-analysis and genetic variants with a MAF > 0.05%, minor allele count > 6, an imputation INFO score > 0.4, MAF +- 20% compared to HRC or 1KG, and a reported p-value with a less than 10% difference with a calculated p-value (from the z-score) in the $-\log_{10}$ scale were included.

Enrichment analysis

To test for enrichment in regions harboring genes differentially expressed during labor in single-cells from myometrium [1], we calculated LD scores (European individuals from phase 3 of the 1000 Genomes project) for sets of genes differentially expressed at labor (± 100 kb) for each cell type separately and for the overall set of genes differentially expressed in the myometrium. In the manuscript, we report exclusively the analysis performed on the overall set of genes, given that the -log10(p-value) for enrichment was a linear function of the number of differentially expressed genes, which ranged from 2 to >3000 (Supplementary Fig. 16). In this context, it is unwise to compare the enrichment in the different cell types. Stratified LD-score regression was run together with the baseline model mentioned above.

Resolving effect origin

In Icelandic data, parental alleles were inferred by combining long-range phasing, genealogy and a maximum likelihood estimation [2–4]. All other cohorts were phased using SHAPEIT2 [5], except HUNT which was phased using Eagle v2.3 [6]. The two methods

use a hidden Markov model in combination with information from genetic relatives to refine phase calls and assign parents to haplotypes. Reliability of all these methods is considered to be high, particularly when large amounts of identical-by-descent segments are present (i.e., when parent-offspring data is available).

Sample size was as follows: 104,962 parent-offspring trios from Iceland with at least one genotyped individual, 17,024 parent-offspring trios from the MoBa cohort, 5,122 parent-offspring trios from the HUNT cohort, and 9,725 mother-child duos from the Avon Longitudinal Study of Parents and Children (ALSPAC), Finnish birth data set (FIN), the Danish National Birth Cohort (DNBC), the Genomic and Proteomic Network for Preterm Birth Research (GPN) and the Hyperglycemia and Adverse Pregnancy Outcome (HAPO). The analysis of the Icelandic data was done on 104,962 parent-offspring trios with at least one genotyped individual. This includes 18,165 fully genotyped trios, 5,208 with only child and mother and 1,875 with only child and father genotyped, 40,182 with both parents genotyped but not the child, and 1,627, 24,965 and 12,868 with only child, mother or father genotyped, respectively.

Colocalization

For each tested locus, this information is summarized in the posterior probability of five hypotheses. Given phenotypes A and B, in a specific locus:

- No association for any of the two phenotypes
- Association with phenotype A but not with phenotype B
- Association with phenotype B but not with phenotype A
- Association with both phenotypes, association driven by SNPs in low LD
- Association with both phenotypes, association driven by SNPs in close LD

The last two hypotheses explicitly model an association with both phenotypes at the same locus, but in the former, the leading SNPs are not shared (i.e., lead variants for the two phenotypes are in low LD) and in the other the leading SNPs are shared (i.e., lead

variants for the two phenotypes are in close LD), despite not knowing which one is the causal one.

Female reproductive traits

To obtain GWAS estimates for preterm delivery independent of the number of live births, we split the cohorts into two groups and then meta-analyzed per strata: on one side, cohorts based on a random pregnancy per mother (the probability of having at least one preterm delivery is not affected by the number of previous or subsequent deliveries) and cohorts with whole reproductive history of a woman (i.e., cohorts using life-time ICD codes or with data on > 1 pregnancy for the same mother).

Locus pleiotropy at 3q21

After identifying locus pleiotropy between the maternal effect on gestational duration and the fetal only effect on birth weight at the *ADCY5* gene region, we set out to investigate differences between the two top SNPs in their colocalization with other traits. Phenome-wide colocalization for the two regions (defined as 1.5Mb around the index SNP) was performed using summary statistics from FinnGen (data freeze 5) and Pan UK Biobank data (https://pan.ukbb.broadinstitute.org, in subjects of recent European ancestry). We included all phenotypes available from FinnGen (n traits= 2,803), while for Pan UK Biobank, we reduced it to summary statistics with an estimated heritability >0.01 and that were labeled as biomarkers, continuous trait or ICD-10 codes (n traits = 832). Given the exploratory nature of this analysis, despite using 3,635 phenotypes, we used a lenient posterior probability of colocalization (>= 0.75).

Gestational duration and preterm delivery polygenic scores

QC of Training Genotypes Dataset

From the training cohort, genotypes were excluded if they had a minor allele frequency of less than 0.01, info scores < 0.7, a Hardy–Weinberg equilibrium less than 1.0×10^{-6} and genotype call rates less than 0.01.

Polygenic Score Calculation

LDpred2 was used for the calculation of the polygenic scores [7]. As we wanted to include the X chromosome in the polygenic score, we mapped the genotypes to the genetic map taken from Bolt-LMM [8]. Polygenic scores were calculated for a range of models using a grid of hyperparameter values; proportion of causal SNPs from 10^{-5} to 1 for 21 values, and proportions of heritability of 0.7, 1, and 1.4, calculated from a constrained LD-score regression. LDpred2 also uses a third hyperparameter that allows for sparse effect size estimates (i.e. some effects are exactly 0). This resulted in a total of 126 combinations of hyperparameter values for the range of grid models [7]. The variance explained was used to decide which of the grid models was the most appropriate polygenic score.

We found the polygenic score (with ten principal components and adjusted for genotyped batch) that utilised the hyperparameters of proportion of causal SNPs of 0.0032, 0.7 of the heritability, and did not allow for sparse effect size estimates, was the most appropriate for the training cohort. This model accessed weighted betas from 1,123,366 variants to explain 2.3% of the variability in the testing sample. We then extracted the weighted betas for each variant from this model to be used in the polygenic score validation.

Latent causal variable analysis

We used latent causal variable analysis to distinguish (partial) causation from genetic correlation [9]. For this, we used the traits that were genetically correlated with gestational duration or preterm delivery (birth weight or sex-hormones). In the LCV model, a latent variable mediates the genetic correlation between two phenotypes. The genetic causality proportion, which quantifies the proportion of the genetic correlation that is due to causality, is then estimated using mixed fourth moments. Whenever GCP p-values were significant, we defined GCP>= 0.6 between two traits as evidence of full or nearly full genetic causality, and GCP< 0.6 as evidence of limited partial causal implication.

Multi-trait conditional analysis

GCTA was used to perform bi-directional multi-trait COJO (mtCOJO) [10] analysis using summary statistics. The gestational duration GWAS was conditioned on the birth weight GWAS and vice-versa, using birth weight summary statistics from the largest GWAS meta-analysis of birth weight [11]. We did not condition on the fetal effects on gestational duration due to a lack of power in the fetal GWAS [12]. We obtained birth weight summary statistics from four different GWAS within the EGG Consortium: using the maternal genome - offspring birth weight, the fetal genome - own birth weight and using a weighted linear model to adjust the GWAS of offspring birth weight by the fetal genome, and the GWAS of own birth weight by the maternal genome.

To select variants with suggestive association with maternal or fetal effects on birth weight, we first split the genome into approximately LD-independent regions, and from each region, selected the variant with the lowest p-value (p-value < 5×10^{-6}). For each of these suggestive variants (87 maternal and 108 fetal), we calculated the relative difference in effect size before and after conditioning as follows,

$$\Delta\beta = \frac{\beta_{conditioned} - \beta_{non-conditioned}}{\beta_{non-conditioned}}$$

A negative $\Delta\beta$ suggests that adjusting for gestational duration reduces the effect size.

To test for robustness of results, we repeated the same analysis for genome-wide significant variants classified as having a maternal only effect on birth weight (n = 31) or a fetal only effect on birth weight (n = 62) [11]. For such variants, we applied the same method as above, multi-trait COJO analysis, and a linear regression model with and without adjusting for gestational duration in individual level data from Iceland (genetic dosage, n mothers = 32,511 and n fetuses = 16,387) and Norway (MoBa, parental transmitted and non-transmitted alleles, n = 21,060 parent-offsprings). For individual level data, we fitted the following two linear models:

$$birth\,weight = SNP + covariates$$

$$birth\,weight = SNP + gestational\,duration + covariates$$

Where SNP is the genotype dosage in Icelandic data for the maternal genome or the fetal genome, and the maternal non-transmitted allele and paternal transmitted allele for maternal and fetal SNPs, respectively. Covariates included the first six principal components and batch for MoBa data. By employing different data sets, and different genetic information (dosage vs parental transmitted / non-transmitted alleles), allowed us to provide further evidence and robustness in our findings.

For all these analyses, we estimated statistical significance of differences between the effect estimates from the two models applying a paired Wilcoxon rank-sum test.

We estimated the heritability of the birth weight GWAS before and after conditioning for gestational duration using LD-score regression [13]. This method estimates polygenic heritability as the variance explained by common genetic variants (autosomal MAF>= 0.01). To test the significance of differences between heritability estimates before and after conditioning, we calculated a z-scored as follows [14],

$$z\,score = \frac{(\beta 1 - \beta 2)}{\sqrt{(se1^2 + se2^2)}}$$

where β1 is the non-conditioned estimate, β2 the conditioned estimate, and $se1$ and $se2$ the standard errors for the non conditioned and conditioned estimates, respectively.

Mendelian randomization

In a two-sample mendelian randomization analysis of gestational duration and birth weight, birth weight was instrumented using summary statistics from a previous GWAS of offspring's birth weight with minimal adjustment by gestational duration (< 15% of samples) [11]. While there is a recent publication on fetal growth [2], this analysis was largely adjusted for gestational duration (> 40% of samples). To avoid confounding due to the correlation between the maternal and fetal genomes, we used summary statistics derived using a weighted linear model [11]. This allowed us to obtain quasi-unbiased estimates for the fetal effects on birth weight (adjusting for the maternal effect):

$$\beta_{fetal\,adjusted} = -\frac{2}{3}\beta_{maternal\,unadjusted} + \frac{4}{3}\beta_{fetal\,unadjusted}$$

and for the maternal effects on birth weight (adjusting for the fetal effect):

$$\beta_{maternal\ adjusted} = -\frac{2}{3}\beta_{fetal\ unadjusted} + \frac{4}{3}\beta_{maternal\ unadjusted}$$

In all two-sample Mendelian randomization analyses, and to obtain a causal estimate, we performed an inverse-variance weighted analysis with standard errors calculated using the delta method [15]. We assessed the impact of horizontal pleiotropy on the causal estimate with MR-Egger regression. The intercept was used to determine whether the average pleiotropic effect is not statistically different from zero (p-value > 0.100). In such cases, the inverse-variance weighted method estimate is a consistent estimate of the causal effect [16]. Whenever the MR-Egger intercept is significantly different from 0, we report the estimate from the MR-Egger analysis. Both inverse-variance weighted method and MR-Egger regression were performed on R using the MendelianRandomization package [17].

Evolutionary analysis

The sequence based evolutionary measures used in this method include: 1) Beta Score which detects balanced polymorphisms to infer balancing selection [18], 2) ARGWEAVE uses ancestral recombination graphs to infer the evolutionary origin of regions [19], 3) GERP uses sequence conservation to infer positive and negative selection [20], 4) LINSIGHT uses sequence conservation to infer positive and negative selection [21], 5) phastCONS100 uses sequence conservation to infer positive and negative selection, 6) PhyloP uses substitution rate to infer positive and negative selection, 7) iES uses haplotype homozygosity to infer positive selection, 8) XPEHH uses haplotype homozygosity to detect population-specific positive selection, and 9) Fst uses population differentiation to infer local adaptation.

Variants from the GWAS that passed a significance threshold (p-value $<1\times10^{-8}$) were clumped into regions using PLINK such that the clumps of variants had an R^2 >0.9 and were within 500 kb. We then obtained 5,000 control variants matched on variant count, LD structure and minor allele frequency. The evolutionary metrics were obtained for all variants, and the maximum value was extracted for analysis. Finally, the evolutionary

metrics were also obtained for the control variants and further used to create a background distribution. Then a z-score and p-value were produced for each experimental genomic region compared to its unique background distribution.

Software

The following software were used for data analysis:

- R version 4.1.1 (https://www.r-project.org) with packages: data.table 1.14.0, dplyr 1.0.7, tidyr 1.1.3, scales 1.1.1, knitr 1.33, cowplot 1.1.0, ggrepel 0.9.1, showtext 0.9_3, tidyverse 1.3.1, fmsb 0.7.1, ggtern 3.3.5, MendelianRandomization 0.5.1, gridextra 2.3, dendextend 1.15.1, plyr 1.8.6, ggtree 3.0.1, kableextra 1.3.4, metafor 4.3.0; coloc v3.0;
- Python version 3.7.9 with packages: pandas 1.1.4, numpy 1.19.5, urllib3 1.26.6, scipy 1.5.3;
- Other software: PLINK v1.90b6.6 64-bit (https://www.cog-genomics.org/plink/); PLINK v2.00a2.3LM (https://www.cog-genomics.org/plink/2.0/); METAL - version released on 2011-03-25 (https://genome.sph.umich.edu/wiki/METAL); bcftools 1.9 (https://samtools.github.io/bcftools/bcftools.html); ensembl-vep 106.1 (https://grch37.ensembl.org/Homo_sapiens/Tools/VEP); qctool v2.0.8 (https://www.well.ox.ac.uk/~gav/qctool/); GCTA 1.93.2beta (https://yanglab.westlake.edu.cn/software/gcta/#Overview); LCV (https://github.com/lukejoconnor/LCV, cloned the 24/08/2021); Python 2.7 with the following packages: scipy 0.18, pandas 0.20, numpy 1.16; LDSC v1.0.1 (https://github.com/bulik/ldsc, cloned the 27/05/2020); bedtools v2.29.2 (https://bedtools.readthedocs.io/en/latest/); BOLT-LMM v2.3 (https://alkesgroup.broadinstitute.org/BOLT-LMM/BOLT-LMM_manual.html#x1-5700012); LDpred2 (https://privefl.github.io/bigsnpr/articles/LDpred2.html); SHAPEIT2 (https://mathgen.stats.ox.ac.uk/genetics_software/shapeit/shapeit.html); EAGLE v2.3 (https://alkesgroup.broadinstitute.org/Eagle/#x1-40002.1)

Consortium author lists and affiliations

Danish Blood Donor Study Genomic Consortium

Steffen Andersen[81], Karina Banasik[8], Søren Brunak[8], Kristoffer Burgdorf[28], Maria Didriksen[28], Khoa Manh Dinh[43], Christian Erikstrup[43], Daniel Gudbjartsson[2], Thomas F. Hansen[82], Henrik Hjalgrim[12,83], Gregor Jemec[84], Poul Jennum[85], Pär I. Johansson[28], Margit A. Larsen[28], Susan Mikkelsen[43], Kasper R. Nielsen[86], Mette Nyegaard[61], Sisse R. Ostrowski[28], Ole B. Pedersen[41], Kari Stefansson[2], Hreinn Stefensson[2], Susanne Säkmose[41], Erik Sørensen[28], Unnur Thorsteinsdottir[2], Mie T. Brun[87], Henrik Ullum[54], Thomas Werge[88]

[81]Department of Finance, Copenhagen Business School, Copenhagen, Denmark
[82]Danish Headache Center, Department of Neurology, Copenhagen University Hospital, Rigshospitalet‟ Glostrup, Glostrup, Denmark
[83]Centre for Cancer Research, Danish Cancer Society, Copenhagen, Denmark
[84]Department of Dermatology, Zealand University hospital - Roskilde, Roskilde, Denmark
[85]Department of clinical neurophysiology, University of Copenhagen, Copenhagen, Denmark
[86]Department of Clinical Immunology, Aalborg University Hospital, Aalborg, Denmark
[87]Department of Clinical Immunology, Odense University Hospital, Odense, Denmark
[88]Institute of Biological Psychiatry Mental Health Centre, Sct. Hans, Copenhagen University Hospital ‟ Roskilde, Roskilde, Denmark

Estonian Biobank Research Team

Triin Laisk[6], Reedik Mägi[6], Andres Metspalu[6], Lili Milani[6], Tõnu Esko[6], Mari Nelis[6], Georgi Hudjashov[6]

Early Growth Genetics

Emma Ahlqvist[89], Tarunveer S. Ahluwalia[90,91,92], Jonas Bacelis[93], Robin N. Beaumont[94], Thomas Bond[95,96,97,13], Dorret I. Boomsma[98,99,14], Jonathan P. Bradfield[50,100], Lachlan Coin[101], Cyrus Cooper[102], Diana L. Cousminer[103,104,105], John A. Curtin[106], Adnan Custovic[107], Felix R. Day[108], Maneka De Silva[70], Paul Elliott[95], Joao Fadista[12], Bjarke Feenstra[12], Romy Gaillard[109], Frank Geller[12], Niels A. Grarup[110], Leif Groop[111,89], Monica Guxens[112,113,114,115], Dexter Hadley[116], Johannes Hebebrand[117,118], O˜yvind Helgeland[15,119,120], Tine B. Henrisksen[121], Anke Hinney[117,118], Joel N. Hirschhorn[122], Marie-France Hivert[123,124,125], Berthold Hocher[126,127,128], John W. Holloway[129], Momoko Horikoshi[130], Jouke-Jan Hottenga[98,131], Stefan Johansson[119,132], Heidi J. Kalkwarf[133], Sailesh Kotecha[134], Zoltan Kutalik[135,136,137], Joan M. Lappe[138], Alexandra M. Lewin[139], Cecilia M. Lindgren[16,140], Reedik Mägi[6], Per Magnus[141], Nina S. McCarthy[142], Camilla S. Morgen[143], Louis J. Muglia[144,145,146,147], Ronny Myhre[148], Ioanna Ntalla[149], Sharon E. Oberfield[150], Emily Oken[123], Beate St Pourcain[151,152], Rashmi B. Prasad[89,153], Rebecca M. Reynolds[154], Rebecca C. Richmond[152,14], Alina Rodriguez[155,156], Rany Salem[157,158,159,160], Theresia M. Schnurr[110], John A. Shepherd[161], Angela Simpson[106], Line Skotte[12], Eric A. Steegers[162], Jordi Sunyer[112,163,114,164], Elisabeth Thiering[165,166], Jessica Tyrrell[94,167], Marc Vaudel[3,15], Carol A.

Wang[168,169], Nicole M. Warrington[101,97,11], William J. Watkins[134], H-Erich Wichmann[170,171,172], Babette S. Zemel[173], Ge Zhang[174,145,146], Andre G. Uitterlinden[175], Andrew P. Morris[176], Andrew T. Hattersley[177], Gonneke Willemsen[178], Allan Linneberg[179], Antje Kärner[180,181,182], Bo Korner[1,15], Christine Power[59], Cornelia van Dujin[183,184], Craig E. Pennell[168,169], Deborah A Lawlor[152,185], David Evans[101,186,152], Dennis Mook-Kanamori[187,188], George Dedoussis[189], Ellen A. Nohr[190], Eleftheria Zeggini[191,192], Elina Hypponen[31,32], Elisabeth Widen[193], M G. Hayes[194,195,196], Struan F. Grant[197,198,199,200,50], Hanieh Yaghootkar[201,202,203], Hakon Hakonarson[204], Inga Prokopenko[205,206,207], Janine Felix[208,209], Jeff Murray[210], James F. Wilson[211], Jun Liu[212], Joachim Heinrich[213], Johan Eriksson[214,215,216], John Perry[217], Jonas Bacelis[1,218], Klaus Bannelykke[90], Ken Ong[217,219], Sailesh Kotecha[134], Kalliope Panoutsopoulou[220], Linda S. Adair[221], Marjo-Riitta Järvelin[222,223,224,225,226], Marie Standl[165], Mark I. McCarthy[227], Mads Melbye[228,229,37,230], Karen L. Mohlke[231], Mustafa Atalay[232], Nic J. Timpson[13], Ni Wang[233], Olli T. Raitakari[234,235,236], Oluf Pedersen[237], Pal R. Njölstad[238,79], Rachel Freathy[239], Rebecca Reynolds[240], Ronald C. Ma[241,242,243], Seang Mei Saw[244,245], David Strachan[246], Yik Ying Teo[247,248], Sylvain Sebert[249], Tim Frayling[250], Tanja Vrijkotte[251], Terho Lehtimaki[252,253,254], Thorkild I. Sorensen[255], Timo A. Lakka[256], Torben Hansen[257], Vincent Jaddoe[208,209], Virpi Lindi[232], Bill Lowe[258], Mariona Bustamante[112,163,114], Jose Ramon Bilbao[259,260,261], Martine Vrijheid[112,163,114], Pol Sole-Navais[1], Izzuddin Aris[123], Sara E. Stinson[257], Jens-Christian Holm[262], Anni Heiskala[263], Justiina Ronkainen[263], Christopher Flatley[93], Alexessander Couto-Alves[264], Juha Mykkanen[234,235], William Schierding[265], Alice E. Hughes[266], Joseph T. Glessner[267,268], Marika A. Kaakinen[269,270,271], Irfahan Kassam[272], Triinu Peters[117], William E. Copeland[273], Xiaoping Wu[12], Jeffrey J. Beck[274,275], Joshua Fisher[276], Yee-Ming Chan[277,160,278], Gunn-Helen Moen[279,23,280,281], Jia Zhu[282], Roseann E. Peterson[283], Brandon Lim[94], Sonia Anand[284,285,286], Amel Lamri[284,285], Ehsan Motazedi[251], Leo-Pekka Lyytikanen[252,253,254], Katja Pahkala[234,235,287], Harri Niinikoski[234,235,288], Aino-Maija Eloranta[289], Sami Heikkinen[232]

[89]Department of Clinical Sciences, Diabetes and Endocrinology, Lund University Diabetes Centre, Malmo, Sweden
[90]COPSAC, Copenhagen Prospective Studies on Asthma in Childhood, Herlev and Gentofte Hospital, University of Copenhagen, Copenhagen, Denmark
[91]Steno Diabetes Center Copenhagen, Herlev, Denmark
[92]The Bioinformatics Center, Department of Biology, University of Copenhagen, Copenhagen, Denmark
[93]Department of Obstetrics and Gynecology, Sahlgrenska University Hospital, Gothenburg, Sweden
[94]Institute of Biomedical and Clinical Science, University of Exeter Medical School, University of Exeter, Royal Devon and Exeter Hospital, Exeter, United Kingdom
[95]Department of Epidemiology and Biostatistics, School of Public Health, Imperial College London, London, United Kingdom
[96]MRC-PHE Centre for Environment and Health, School of Public Health, Imperial College London, London, United Kingdom.
[97]University of Queensland Diamantina Institute, The University of Queensland, Brisbane, Australia
[98]Netherlands Twin Register, Department of Biological Psychology, Vrije University, Amsterdam, the Netherlands
[99]Amsterdam Reproduction & Development (AR&D) research institute, the Netherlands
[100]Quantinuum Research LLC, San Diego, United States

[101]Institute for Molecular Bioscience, University of Queensland, Brisbane, Australia
[102]MRC Lifecourse Epidemiology Unit, Faculty of Medicine, University of Southampton, Southampton, United Kingdom
[103]Division of Human Genetics, Children’s Hospital of Philadelphia, Philadelphia, PA, United States.
[104]Department of Genetics, University of Pennsylvania, Philadelphia, PA, United States
[105]Center for Spatial and Functional Genomics, Childrenâ ™s Hospital of Philadelphia, Philadelphia, PA, United States
[106]Division of Immunology, Immunity to Infection and Respiratory Medicine, School of Biological Sciences, The University of Manchester, Manchester Academic Health Science Centre, and Manchester University NHS Foundation Trust, Manchester, United Kingdom
[107]National Heart and Lung Institute, Imperial College London, United Kingdom
[108]MRC Epidemiology Unit, University of Cambridge School of Clinical Medicine, Cambridge, United Kingdom
[109]Department of Epidemiology, Erasmus MC University Medical Center, Rotterdam, the Netherlands
[110]Novo Nordisk Foundation Center for Basic Metabolic Research, Section of Metabolic Genetics, Faculty of Health and Medical Sciences, University of Copenhagen, Copenhagen, Denmark
[111]Institute for Molecular Medicine, Finland (FIMM), University of Helsinki, Helsinki, Finland
[112]ISGlobal, Institute for Global Health, Barcelona, Spain
[113]CIBER de Epidemiologia y Salud Publica (CIBERESP), Instituto de Salud Carlos III, Madrid, Spain
[114]Universitat Pompeu Fabra (UPF), Barcelona, Spain
[115]Department of Child and Adolescent Psychiatry/Psychology, Erasmus MC, University Medical Centre, Rotterdam, The Netherlands
[116]Department of Pediatrics, University of California San Francisco School of Medicine, San Francisco, United States
[117]Department of Child and Adolescent Psychiatry, Psychosomatics and Psychotherapy, University Hospital Essen, University of Duisburg-Essen, Essen, Germany
[118]Center for Translational Neuro- and Behavioural Sciences, University Hospital Essen, Essen, Germany
[119]KG Jebsen Center for Diabetes Research, Department of Clinical Science, University of Bergen, Bergen, Norway
[120]Department of Pediatrics, Haukeland University Hospital, Bergen, Norway
[121]Department of Clinical Medicine - Department of Paediatrics, Aarhus University Hospital, Aarhus N, Denmark
[122]Broad Institute of MIT and Harvard, Cambridge, United States
[123]Department of Population Medicine, Harvard Pilgrim Health Care Institute, Harvard Medical School, Boston, MA, United States
[124]Diabetes Center, Massachusetts General Hospital, Boston, MA, United States
[125]Department of Medicine, Universite de Sherbrooke, Sherbooke, Canada
[126]Institute of Nutritional Science, University of Potsdam, Nuthetal, Germany
[127]The First Affiliated Hospital of Jinan University, Guangzhou, China
[128]Department of Medicine Nephrology, Medical Faculty, Mannheim Heidelberg University, 68167 Mannheim, Germany
[129]Human Development & Health, Faculty of Medicine, University of Southampton, Southampton, United Kingdom
[130]RIKEN, Centre for Integrative Medical Sciences, Laboratory for Genomics of Diabetes and Metabolism, Yokohama, Japan
[131]Amsterdam Public Health, Amsterdam, the Netherlands
[132]Center for Medical Genetics and molecular Medicine, Haukeland University Hospital, Bergen, Norway

[133]Division of Gastroenterology, Hepatology and Nutrition, Cincinnati Children's Hospital Medical Center, Cincinnati, United States
[134]Department of Child Health, School of Medicine, Cardiff Univeristy, Cardiff, United Kingdom
[135]Institute of Primary Care and Public Health, University of Lausanne, Lausanne, Switzerland
[136]Department of Computational Biology, University of Lausanne, Lausanne, Switzerland
[137]Swiss Institute of Bioinformatics, Lausanne, Switzerland
[138]Division of Endocrinology, Department of Medicine, Creighton University, Omaha, United States
[139]Department of Medical Statistics, London School of Hygiene and Tropical Medicine, London, United Kingdom
[140]Li Ka Shing Centre for Health Information and Discovery, The Big Data Institute, University of Oxford, Oxford, United Kingdom
[141]Norwegian Institute of Public Health, Oslo, Norway
[142]Centre for Genetic Origins of Health and Disease (GOHaD), The University of Western Australia, Crawley, Australia
[143]Department of Public Health, Section of Epidemiology, Faculty of Health and Medical Sciences, University of Copenhagen, Copenhagen, Denmark
[144]Burroughs Wellcome Fund, Research Triangle Park and Department of Pediatrics, Cincinnati Children's Hospital Medical Center and University of Cincinnati College of Medicine, Cincinnati, OH, United States
[145]March of Dimes Prematurity Research Center Ohio Collaborative, Cincinnati, United States
[146]Human Genetics Division, Cincinnati Childrenâ ™s Hospital Medical Center, Cincinnati, United States
[147]President, Burroughs-Wellcome Fund
[148]Department of Genes and Environment, Division of Epidemiology, Norwegian Institute of Public Health, Oslo, Norway
[149]William Harvey Research Institute, Barts and the London School of Medicine and Dentistry, Queen Mary University of London, London, United Kingdom
[150]Division of Pediatric Endocrinology, Diabetes, and Metabolism, Department of Pediatrics, Columbia University Medical Center, New York, United States
[151]Max Planck Institute for Psycholinguistics, Nijmegen, the Netherlands
[152]MRC Integrative Epidemiology Unit at the University of Bristol, Bristol, United Kingdom
[153]Institute of Molecular Medicine, Helsinki, Finland
[154]Centre for Cardiovascular Science, Queen's Medical Research Institute, University of Edinburgh, Edinburgh, United Kingdom
[155]Department of Epidemiology and Biostatistics, MRC-PHE Centre for Environment & Health, School of Public Health, Imperial College London, London, United Kingdom
[156]Department of Psychology, Mid Sweden University, Ostersund, Sweden
[157]Herbert Wertheim School of Public Health, University of California San Diego, La Jolla, CA United States
[158]Department of Genetics, Harvard Medical School, Boston, MA, United States
[159]Center for Basic and Translational Obesity Research, Boston Children's Hospital, Boston, MA, United States
[160]Program in Medical and Population Genetics, Broad Institute of Harvard and MIT, Cambridge, United States
[161]Department of Radiology, University of California San Francisco, San Francisco, United States
[162]Department of Obstetrics and Gynecology, Erasmus MC, University Medical Center, Rotterdam, the Netherlands
[163]CIBER de Epidemiologia y Salud Publica (CIBERESP), Madrid, Spain
[164]IMIM (Hospital del Mar Medical Research Institute), Barcelona, Spain

[165]Institute of Epidemiology, Helmholtz Zentrum Munchen - German Research Center for Environmental Health, Neuherberg, Germany
[166]Division of Metabolic and Nutritional Medicine, Dr. von Hauner Children's Hospital, University of Munich Medical Center, Munich, Germany
[167]European Centre for Environment and Human Health, University of Exeter, Truro, United Kingdom
[168]School of Medicine and Public Health, University of Newcastle, Newcastle, NSW, Australia
[169]Hunter Medical Research Institute, Newcastle, NSW, Australia
[170]Institute of Medical Informatics, Biometry and Epidemiology, Chair of Epidemiology, Ludwig Maximilians University, Munich, Germany
[171]Helmholtz Center Munich, Institute of Epidemiology, Neuherberg, Germany
[172]Institute of Medical Statistics and Epidemiology, Technical University Munich, Munich, Germany
[173]Division of Gastroenterology, Hepatology and Nutrition, The Children´s Hospital of Philadelphia, Philadelphia, United States
[174]Division of Human Genetics, Cincinnati Children's Hospital Medical Center, Center for Prevention of Preterm Birth, Perinatal Institute, Cincinnati Children's Hospital Medical Center, Department of Pediatrics, University of Cincinnati College of Medicine, Cincinnati, United States
[175]Department of Internal Medicine, Erasmus Medical Centre, Rotterdam, The Netherlands
[176]Centre for Genetics and Genomics Verusus Arthritis, Centre for Musculoskeletal Research, The University of Manchester, Manchester, United Kingdom
[177]University of Exeter Medical School, College of Medicine and Health, University of Exeter, Exeter, United Kingdom
[178]Netherlands Twin Register, Department of Biological Psychology, Vrije University, Amsterdam, Netherlands
[179]Center for Clinical Research and Prevention, Bispebjerg and Frederiksberg Hospital, Copenhagen, Denmark and Department of Clinical Medicine, Faculty of Health and Medical Sciences,University of Copenhagen, Copenhagen, Denmark
[180]University of Leipzig, Medical Faculty, Dept. of Women and Child Health, Pediatric Research Center, Leipzig, Germany
[181]Helmholtz Institute for Metabolic, Obesity and Vascular Research (HI-MAG) of the Helmholtz Zentrum Munchen at the University of Leipzig and University Hospital Leipzig, Leipzig, Germany
[182]LIFE Child, University of Leipzig, Medical Faculty, LIFE “Leipzig Research Center for Civilization Diseases, Leipzig, Germany,
[183]Department of Epidemiology, Erasmus MC University Medical Center, Rotterdam, The Netherlands
[184]Department of Public Health, Oxford University, Oxford, United Kingdom
[185]Population Health Science, Bristol Medical School, Bristol University, United Kingdom
[186]University of Queensland Diamantina Institute, University of Queensland, Brisbane, Australia
[187]Department of Clinical Epidemiology, Leiden University Medical Center, 2333 ZA Leiden, The Netherlands
[188]Department of Public Health and Primary Care, Leiden University Medical Center, 2333 ZA Leiden, The Netherlands
[189]Department of Nutrition and Dietetics, Harokopio University of Athens, Athens, Greece.
[190]Department of Clinical Research, Research Unit for Obstetrics and Gynecology, University of Southern Denmark, Odense, Denmark
[191]Institute of Translational Genomics, Helmholtz Zentrum Munchen â “ German Research Center for Environmental Health, 85764 Neuherberg, Germany
[192]Technical University of Munich (TUM) and Klinikum Rechts der Isar, TUM School of Medicine, Ismaninger Str. 22, 81675 Munich, Germany
[193]Institute for Molecular Medicine Finland (FIMM), HiLIFE, University of Helsinki, Helsinki, Finland

[194]Division of Endocrinology, Metabolism, and Molecular Medicine, Department of Medicine, Northwestern University Feinberg School of Medicine, Chicago, IL United States
[195]Center for Genetic Medicine, Northwestern University Feinberg School of Medicine, Chicago, IL United States
[196]Department of Anthropology, Northwestern University, Evanston, IL United States
[197]Division of Human Genetics, The Children's Hospital of Philadelphia, Philadelphia, United States
[198]Division of Endocrinology and Diabetes, The Children's Hospital of Philadelphia, Philadelphia, United States
[199]Department of Pediatrics, University of Pennsylvania Perelman School of Medicine, Philadelphia, United States
[200]Department of Genetics, University of Pennsylvania Perelman School of Medicine, Philadelphia, United States
[201]Genetics of Complex Traits, University of Exeter Medical School, University of Exeter, Royal Devon & Exeter Hospital, Exeter, U.K
[202]Research Centre for Optimal Health, School of Life Sciences, University of Westminster, London, U.K
[203]Department of Life Sciences, Centre for Inflammation Research and Translational Medicine, Brunel University London, London, U.K.
[204]Childrenâ ™s Hospital of Philadelphia, Leonard Madlyn Abramson Research Center, Philadelphia, PA, United States
[205]Department of Clinical & Experimental Medicine, University of Surrey, Guildford, United Kingdom
[206]UMR 8199 - EGID, Institut Pasteur de Lille, CNRS, University of Lille, F-59000 Lille, France.
[207]Institute of Biochemistry and Genetics, Ufa Federal Research Centre, Russian Academy of Sciences, Ufa, Russian Federation
[208]The Generation R Study Group, Erasmus MC, University Medical Center Rotterdam, Rotterdam, the Netherlands
[209]Department of Pediatrics, Erasmus MC, University Medical Center Rotterdam, Rotterdam, the Netherlands
[210]Dept of Pediatrics, University of Iowa, Iowa City, IA 52240 United States
[211]Centre for Global Health Research, Usher Institute, University of Edinburgh, Teviot Place, Edinburgh, EH8 9AG, Scotland
[212]Nuffield Department of Population Health, Univeristy of Oxford, Oxford, United Kingdom
[213]Institute and Clinic for Occupational, Social and Environmental Medicine, University Hospital, LMU Munich, Munich, Germany
[214]Department of General Practice and Primary Health Care, University of Helsinki, FolkhÃ¤lsan Research Center, Helsinki, Finland
[215]National University Singapore, Yong Loo Lin School of Medicine, Human Potential Translational Research Programme and Department of Obstetrics and Gynecology, Singapore, Singapore
[216]Singapore Institute for Clinical Sciences (SICS), Agency for Science, Technology and Research (A*STAR), Singapore
[217]MRC Epidemiology Unit, Institute of Metabolic Science, University of Cambridge School of Clinical Medicine, Cambridge, United Kingdom
[218]Region Västra Götaland, Sahlgrenska University Hospital, Department of Obstetrics and Gynecology, Gothenburg, Sweden
[219]Department of Paediatrics, University of Cambridge School of Clinical Medicine, Cambridge, United Kingdom
[220]Wellcome Trust Sanger Institute, Wellcome Genome Campus, Hinxton, Cambridgeshire, United Kingdom
[221]Department of Nutrition, University of North Carolina, Chapel Hill, NC, United States

[222]Institute of Health Sciences, University of Oulu, Oulu, Finland
[223]Biocenter Oulu, University of Oulu, Oulu, Finland
[224]Department of Epidemiology and Biostatistics, MRC Health Protection Agency (HPE) Centre for Environment and Health, School of Public Health, Imperial College London, United Kingdom
[225]Department of Children and Young People and Families, National Institute for Health and Welfare, Oulu, Finland
[226]Unit of Primary Care, Oulu University Hospital, Oulu, Finland
[227]Wellcome Centre for Human Genetics, Univ of Oxford, Oxford, United Kingdom (for papers submitted before end 2022); after that, please contact me. Also pls include (Current address: Genentech, 1 DNA Way, South San Francisco, CA 94080) as a footnote
[228]Department of Clinical Medicine, Universityof Copenhagen, Copenhagen, Denmark
[229]Department of Medicine, Stanford UniversitySchool of Medicine, Stanford, California, United States
[230]K.G. Jebsen Center for Genetic Epidemiology,Norwegian University of Science andTechnology, Trondheim, Norway
[231]Department of Genetics, University of North Carolina, Chapel Hill, NC, United States
[232]Institute of Biomedicine, School of Medicine, University of Eastern Finland, Kuopio Campus, Finland
[233]COPSAC Copenhagen Prospective Studies on Asthma in Childhood Copenhagen University Hospital, Herlev-Gentofte
[234]Centre for Population Health Research, University of Turku and Turku University Hospital, Turku, Finland
[235]Research Centre of Applied and Preventive Cardiovascular Medicine, University of Turku, Turku, Finland
[236]Department of Clinical Physiology and Nuclear Medicine, Turku University Hospital, Turku, Finland
[237]Novo Nordisk Foundation Center for Basic Metabolic Research, Faculty of Health and Medical Sciences, University of Copenhagen, Copenhagen, Denmark
[238]Mohn Center for Diabetes Precision Medicine, Department of Clinical Science, University of Bergen, NO-5020 Bergen, Norway
[239]Institute of Biomedical and Clinical Science, College of Medicine and Health, University of Exeter, Barrack Road, Exeter, Devon EX2 5DW, United Kingdom
[240]Centre for Cardiovascular Science, Queen ™s Medical Research Institute, University of Edinburgh, Edinburgh, United Kingdom
[241]Department of Medicine and Therapeutics, The Chinese University of Hong Kong
[242]Li Ka Shing Institute of Health Sciences, The Chinese University of Hong Kong, Hong Kong, China
[243]Hong Kong Institute of Diabetes and Obesity, The Chinese University of Hong Kong, Hong Kong, China
[244]Singapore Eye Research Institute, Singapore
[245]Yong Loo Lin School of Medicine, National University of Singapore, Singapore
[246]Population Health Research Institute, St Georgea ™s, University of London, London, United Kingdom
[247]Saw Swee Hock School of Public Health, National University of Singapore and National University Health System, Singapore, Singapore
[248]National University of Singapore, Singapore, Singapore
[249]Research Unit of Population Health, Faculty of Medicine, University of Oulu, Oulu, Finland
[250]Genetics of Complex Traits, College of Medicine and Health, University of Exeter, Exeter, United Kingdom,
[251]Department of Public and Occupational Health, Amsterdam University Medical Center, University of Amsterdam, Amsterdam

[252]Department of Clinical Chemistry, Fimlab Laboratories, Tampere 33520, Finland
[253]Department of Clinical Chemistry, Finnish Cardiovascular Research Center - Tampere, Faculty of Medicine and Health Technology, Tampere University, Tampere 33014, Finland
[254]Department of Cardiology, Heart Center, Tampere University Hospital, Tampere 33521, Finland
[255]Department of Public Health and Novo Nordisk Foundation Center for Basic Metabolic Research, Faculty of Health and Medical Sciences, University of Copenhagen, Denmark
[256]Institute of Biomedicine, School of Medicine, University of Eastern Finland, Kuopio Campus, Finland; Department of Clinical Physiology and Nuclear Medicine, Kuopio University Hospital, Kuopio, Finland; Foundation for Research in Health Exercise and Nutrition, Kuopio Research Institute of Exercise Medicine, Kuopio, Finland
[257]Novo Nordisk Foundation Center for Basic Metabolic Research, Faculty of Health and Medical Sciences, University of Copenhagen, Denmark
[258]Department of Medicine, Northwestern University Feinberg School of Medicine, Rubloff 12, 420 E. Superior St, Chicago, IL 60611, United States
[259]Faculty of Medicine and Nursing, Department of Genetics, Physical Anthropology and Animal Physiology, University of the Basque Country (UPV/EHU), Leioa, Bizkaia, Spain
[260]Biocruces Bizkaia Health Research Institute, Barakaldo, Bizkaia, Spain
[261]CIBER de Diabetes y Enfermedades Metabolicas Asociadas (CIBEDEM), Madrid, Spain
[262]The Children ™s Obesity Clinic, accredited European Centre for Obesity Management, Department of Pediatrics, Holbak Hospital, Denmark
[263]Center for Life Course Health Research, University of Oulu, Oulu, Finland
[264]School of biosciences and medicine, University of Surrey, United Kingdom
[265]Liggins Institute, University of Auckland, Auckland, NZ
[266]Institute of Biomedical and Clinical Science, University of Exeter Medical School, Exeter, United Kingdom
[267]Department of Pediatrics, Children ™s Hospital of Philadelphia, 3401 Civic Center Blvd, Philadelphia, PA 19104, United States.
[268]Department of Pediatrics, Division of Human Genetics, Perelman School of Medicine, 3400 Civic Center Blvd, Philadelphia, PA 19104, United States.
[269]Department of Clinical and Experimental Medicine, University of Surrey, Guildford, United Kingdom
[270]People-Centred Artificial Intelligence Institute, University of Surrey, Guildford, United Kingdom
[271]Department of Medicine, Imperial College London, London, United Kingdom
[272]Lee Kong Chian School of Medicine, Nanyang Technological University, Singapore, Republic of Singapore
[273]Department of Psychitry, University of Vermont, Burlington, Vermont
[274]Avera Institute for Human Genetics, Avera McKennan Hospital and University Health Center, Sioux Falls, South Dakota, United States
[275]Department of Psychiatry, University of South Dakota Sanford School of Medicine, South Dakota, United States
[276]School of Medical Science, Menzies Health Institute Queensland, Griffith University Gold Coast Campus, Southport, QLD, Australia
[277]Division of Endocrinology, Boston Childrenâ ™s Hospital, Boston, MA 02115, United States
[278]Department of Pediatrics, Harvard Medical School, Boston, MA 02115, United States
[279]Institute of Molecular Biosciences, The University of Queensland, Brisbane, Australia
[280]K.G. Jebsen Center for Genetic Epidemiology, Department of Public Health and Nursing, Norwegian University of Science and Technology, Trondheim, Norway.
[281]Population Health Science, Bristol Medical School, University of Bristol, United Kingdom.
[282]Division of Endocrinology, Department of Pediatrics, Boston Children's Hospital
[283]Department of Psychiatry and Behavioral Sciences, College of Medicine, State University of New York Downstate Health Sciences University, Brooklyn, New York, United States

[284]Department of Medicine, McMaster University, Hamilton, Ontario, Canada
[285]Population Health Research Institute, Hamilton Health Sciences and McMaster University, Hamilton, Ontario, Canada
[286]Department of Health Research Methods, Evidence, and Impact, McMaster University, Hamilton, Ontario, Canada.
[287]Paavo Nurmi Centre and Unit for Health and Physical Activity, University of Turku, Turku, Finland
[288]Department of Pediatrics and Adolescent Medicine, University of Turku and University Hospital of Turku, Turku, Finland
[289]Institute of Public Health and Clinical Nutrition, School of Medicine, University of Eastern Finland, Kuopio Campus, Finland

Description of participating cohorts

23andMe

Genome-wide summary statistics from a previously published GWAS on gestational duration and preterm delivery were used [22]. Briefly, participants in the research program of 23andMe, a personal genomics and biotechnology company provided written informed consent and answered surveys online according to a human-subjects protocol approved by Ethical and Independent Review Services (www.eandireview.com). Unrelated women of European ancestry self-reported the gestational duration of their first live singleton birth. Women with a medical indication for preterm delivery were excluded. Preterm-birth status was determined on the basis of dichotomization of gestational duration (preterm, <37 weeks; term, ≥37 weeks).
DNA extraction and genotyping were performed by the National Genetics Institute. The analysis was restricted to 43,568 women. Genotype data were imputed against the reference haplotypes of phase 1 of the 1000 Genomes Project [23].

Linear regression was used to estimate genetic associations with gestational duration and logistic regression to test such associations with preterm birth. The maternal age and the top five principal components were included as covariates.

Avon Longitudinal Study of Parents and Children

The Avon Longitudinal Study of Parents and Children (ALSPAC) is a longitudinal birth cohort, which has been described in detail elsewhere [24,25]. In short, pregnant women resident in Avon, UK with expected dates of delivery 1st April 1991 to 31st December 1992 were invited to take part in the study. The initial number of pregnancies enrolled is 14,541. Of these initial pregnancies, there were a total of 14,676 foetuses, resulting in 14,062 live births and 13,988 children who were alive at 1 year of age. Ethical approval for the study was obtained from the Avon Longitudinal Study of Parents and Children

Law and Ethics Committee (IRB# 00003312) and the Local Research Ethics Committees (Bristol and Weston, Southmead, and Frenchay Health Authorities). Written informed consent was obtained from all adult participants in the study. Consent for biological samples has been collected in accordance with the Human Tissue Act (2004). Please note that the study website contains details of all the data that is available through a fully searchable data dictionary and variable search tool (http://www.bristol.ac.uk/alspac/researchers/our-data/).

Born in Bradford

The Born in Bradford (BiB) cohort study was established in 2007 as detailed elsewhere [26]. All women booked for delivery at the Bradford Royal Infirmary are offered an oral glucose tolerance test (OGTT) at 26–28 weeks gestation. On attendance at the OGTT clinic, full consent was obtained for recruitment to BiB, and the woman was invited to complete an interviewer-administered questionnaire. Between March 2007 and November 2010, >80% of the women who attended the OGTT took up the invitation, and we recruited 12,453 women with 13,776 pregnancies to the cohort. Ethics approval has been obtained for the main platform study and was provided by the Bradford Local NHS Research Ethics Committee (ref 06/ Q1202/48).

British 1958 Birth Cohort (1958BC-T1DGC and 1958BC-WTCCC2)

The 1958 British Birth Cohort (1958BC) consists of all born during one week in March 1958 in England, Scotland, and Wales (n=17,638) [27]. Participants have been followed-up from birth and at age 45 years, 11,971 cohort members who had not died or emigrated, were invited to a biomedical assessment. In this survey 9,377 participants took part in clinical assessments and DNA collection. Ethical approval for the 45y survey was obtained from South East Multi-centre Research Ethics Committee (ref. 01/1/44) and the Joint UCL/UCLH Committees on the Ethics of Human Research (Committee A) Ref: 08/H0714/40.

Genome-wide data has been obtained through two sub-studies, both using the 1958BC as a control population. First, 3000 DNA samples were randomly selected and genotyped on the Affymetrix SNP 6.0 platform as part of the Wellcome Trust Case Control Consortium (WTCCC2) [28]. Secondly, 2,592 participants from the 1958BC were used as controls for a Type 1 diabetes case-control study (T1DGC). DNA samples were genotyped through the JDRF/WT Diabetes and Inflammation Laboratory (DIL) using the Illumina Infinium 550K chip [29]. Imputation was done in IMPUTE after quality control. For B58C-WTCCC2 quality control included SNP exclusions (Minor allele frequency (MAF) < 0.01, HWE P-value <1 x 10-20, call rate < 0.98, genotype plate association <1 x 10-5), and sample exclusions (heterozgygosity, call rate, relatedness, non-European ancestry and sex discrepancy). For B58C-T1DGC, criteria for SNP exclusions were MAF < 0.01, HWE

P-value <1 x 10-7, or SNP call rate < 0.95, and sample exclusions were made for heterozygosity, call rate, non-European ancestry and potential sex discrepancy.

Information on gestational duration was collected at the 33-year and 41-year follow-up surveys [30], when cohort members reported whether they had been pregnant and if so, what was the outcome of each pregnancy (miscarriage, abortion, stillbirth, livebirth). For live births, mothers provided information about birth weight. Information on gestational duration was asked by a question whether the child was born at term, or alternatively how many weeks in advance or late.

Children's Hospital of Philadelphia (CHOP)

All subjects were consecutively recruited from the Greater Philadelphia area from 2006 to 2021 at the Children's Hospital of Philadelphia. Our study cohort consisted of children of European ancestry. All these participants had their blood drawn into an 8ml EDTA blood collection tube and were subsequently DNA extracted for genotyping. All subjects were biologically unrelated and were aged less than 18 years old at the time of blood collection. This study was approved by the Institutional Review Board of the Children's Hospital of Philadelphia. Parental informed consent was given for each study participant for both the blood collection and subsequent genotyping.

We performed high throughput genome-wide SNP genotyping, using the Illumina Infinium™ II HumanHap550 and Human610 BeadChip technology (Illumina, San Diego), at the Center for Applied Genomics at CHOP. We used 750ng of genomic DNA to genotype each sample, according to the manufacturer's guidelines.

Samples were genotyped on a combination of the HumanHap 550 version 1, HumanHap 550 Version 3 and 610 Quad SNP chips. The SNPs in common with the three different chip versions used were the basis for all further analyses. The Sanger Imputation Server was used to impute ~40 million SNPs using the HRC 1.1 reference haplotypes.

Danish Blood Donor Study

The Danish Blood Donor Study (DBDS) is a prospective cohort of blood donors of whom the first of 116,000 individuals have been genotyped [31]. This study was performed under the reproductive health protocol (CVK-1805807). The DBDS cohort included 20,577 unique pregnancies identified from the Danish Medical Birth Registry (DMBR, 1,430, 485, 4,794 deliveries <259 days, <238 days, and >294 days, respectively) [32]. The DMBR covers all births since 1973 (both live- and still-births). Multi-fold births and still-births were excluded from the analysis. Furthermore, pregnancies with recorded obstetric complications: placental abruption, placenta previa, pre-eclampsia, eclampsia, polyhydramnios, oligohydramnios, placental insufficiency, cervical insufficiency,

isoimmunization, breech presentation or maternal conditions: diabetes (Types I, II, gestational), hypertension, autoimmune diseases (SLE, RA, IBD, scleroderma), immunodeficiency states (HIV), abnormality of pelvic organs were excluded. Complications and maternal conditions were identified by linkage with the Danish National Patient Registry (DNPR). The DNPR covers all hospital admissions starting from 1977. DBDS samples were genotyped at deCODE Genetics using the Illumina Global Screening Array. Imputation was performed using a Northern European reference panel based on whole-genome sequencing. Quality control filtering required sample call rate of ≥0.98 and principal-component analysis was used to exclude ancestral outliers. We also performed SNP quality control (call rate of ≥0.98 in both cases and controls and HWE P of ≥1 × 10−6 in controls). Association testing was performed using BOLT-LMM [33](v2.3) or SAIGE (v0.36.3.3) [34] for the continuous and binary phenotypes, respectively, including year of birth, year of birth squared, and the first ten principal components.

deCODE

The study was approved by the National Bioethics Committee, Iceland (approval no. VSN-15-169) following evaluation of the Icelandic Data Protection Authority. We have obtained informed consent for all participants in this study who donated samples.
Information on gestational age comes from the Icelandic birth register, which includes 142,447 Icelanders born between 1982 and 2016 [2]. After excluding multiple births and infant deaths, we had information on gestational duration for 61,116 mothers. Mean birth year of mothers was 1969 (s.d. 25.1) and mean age at the birth of first child was 26.6 (s.d. 6.0).

Genotyping and imputation

Genotyping and imputation of the Icelandic samples were performed as previously described [35,36]. In short, we sequenced the whole genomes of 28,075 Icelanders using Illumina technology to a mean depth of at least 10X (median 32X). Genotypes were called using joint calling with the Genome Analysis Toolkit HaplotypeCaller (GATK version 3.4.07) [37]. Genotype calls were improved by using information about haplotype sharing, taking advantage of the fact that all sequenced individuals had also been chip-typed and long-range phased. About 33 million variants that passed the quality threshold were then imputed into 155,250 Icelanders, who had been genotyped with various Illumina SNP chips and their genotypes phased using long-range phasing [3,4]. Using genealogical information, the sequence variants were also imputed into 285,664 of their first- and second-degree relatives [35]. Out of 61,116 mothers included in the study, 37,397 were chip typed.

Association testing

We used logistic regression assuming an additive model in the case-control analysis to test for association between variants and disease, treating disease status as the response and expected genotype counts from imputation as covariates, and using likelihood ratio test to compute P-values. The model also included adjustment for additional covariates, i.e. county of birth, current age or age at death (first and second order terms included), availability of blood sample for the individual and an indicator function for the overlap of the lifetime of the individual with the time span of phenotype collection. To test the association of sequence variants with gestational duration we used a linear mixed model implemented in BOLT-LMM [33]. Prior to association analysis the measurements were adjusted for year of birth and age and, where appropriate, normalized to a standard normal distribution using rank based inverse normal transformation. All the analysis was done using software developed at deCODE genetics [35]. Only variants with imputation info over 0.8 and MAF > 0.01% were included. To account for inflation in test statistics due to cryptic relatedness and stratification, we applied the method of LD score regression [13], using an LD score estimated from the European ancestry samples in the 1000 Genomes Project, to estimate the inflation in the test statistics and adjusted all P-values accordingly.

Danish National Birth Cohort - DNBC

The DNBCGOYACASES, DNBCGOYACONTROLS, and DNBCPTD samples are sub-cohorts nested within the Danish National Birth Cohort (DNBC) [38]. The DNBC is a collection of data on 92,274 pregnant women recruited between 1996 and 2002, from their first antenatal visit to their general practitioner. Women participated in four telephone interviews (16 and 30 weeks gestation and 6 and 18 months after birth). They also provided two blood samples during pregnancy. Information about pregnancy outcomes for the DNBC participants was obtained from the Danish Medical Birth Register [32] and the Danish National Patient Register [39]. Gestational duration in this dataset was determined by a consensus algorithm combining all available information from multiple sources: self-reported date of last menstrual period, self-reported expected delivery date, and gestational age at birth registered in the Medical Birth Register and the National Patient Register.

The participants in the GOYA (Genomics of extremely Overweight Young Adults) study were drawn from the 67,863 women within the DNBC who provided information about prepregnancy BMI, gave birth to a live born infant and provided a blood sample during pregnancy [40]. A case sample of the 3.6% most obese women (DNBCGOYACASES; n=2451) was defined as those with the largest residuals from the regression of BMI on age and parity (all entered as continuous variables). The BMI for these women ranged from 32.6 to 64.4. From the remaining cohort we selected a random sample of similar size (DNBCGOYACONTROLS; n=2450). Genome-wide SNP array data for the GOYA participants were generated using the Illumina Human 610 Quad v1.0 BeadChip. Ethical

approval was obtained from the Regional Scientific Ethical Committee of the Region of Mid Jutland and the study was also approved by the Danish Data Protection Agency.

The Danish National Birth Cohort - Preterm Delivery Study (DNBCPTD) is a case-control study [41] using mother-infant pairs to investigate genetic and environmental influences on spontaneous preterm birth. The study is nested within the DNBC; for case pairs delivery occurred before 37 weeks of gestation and for control pairs delivery occurred in gestational week 39, 40, or 41. Exclusion criteria were multiple deliveries, pregnancy complications such as placental abnormalities, preeclampsia/eclampsia, congenital abnormalities or stillbirth. Individuals were further required to be of Northern European ancestry. Genome-wide genotype data were obtained using the Illumina Human 660W Quad array and generated as part of the Gene Environment Association Studies (GENEVA) consortium [42]. Ethical approval was obtained from the Regional Scientific Ethical Committee of Copenhagen and the study was also approved by the Danish Data Protection Agency.

After genotyping, data from the DNBCGOYACASES, DNBCGOYACTRLS, and DNBCPTD samples were aligned to the forward strand and combined, including only overlapping SNPs between the Illumina Human 610-Quad and Human 660W Quad arrays. Data cleaning and QC steps were based on the following requirements for samples and SNPs to be included: sample missingness rate < 4%, heterozygosity within 3 standard deviations from the mean, variant missingness rate < 2%, minor allele frequency > 1%, A/T and C/G variants excluded, Hardy-Weinberg P value > 1e-06. Furthermore, principal components analysis jointly with data from the Human Genome Diversity Project was used to restrict the analysed sample to individuals of European ancestries, and individuals were excluded if they were related to another participant in the sample (corresponding to an IBD proportion >0.1875). The cleaned data containing 502,119 SNPs was phased using EAGLE v2.3 [6] and imputed to the Haplotype Reference Consortium release 1.1 [43] panel using Minimac3 [44]. After imputation, association testing was carried out in the DNBCGOYACASES (n=1610), DNBCGOYACTRLS (n=1912), and DNBCPTD (n=2226) cohorts separately using SNPTEST 2.5.2 [45].

For analyses of parental transmitted and non-transmitted data, data downloaded from the Database of Genotypes and Phenotypes (dbGaP) (phs000103.v1.p1) was used.

Estonian Genome Center of the University of Tartu

The Estonian Genome Center of the University of Tartu (EGCUT) is a population-based biobank with 51,515 participants in the first wave of data collection [46]. Data on diagnoses is obtained via periodical linking of the EGCUT dataset to national electronic databases and registries [46]. All EGCUT participants have signed a broad informed

consent form and the study was conducted under the approval 234T-12 issued by the Ethics Review Committee of the University of Tartu.

Exeter Family of Childhood Health (EFSOCH)

The EFSOCH study [47] is a prospective study of children born between 2000 and 2004, and their parents, from a geographically defined region of Exeter, UK. All women gave informed consent and ethical approval was obtained from the local review committee. Gestational age at delivery was calculated based on the last menstrual period, or when that was unreliable or unavailable, dating by ultrasound scan was used.

Maternal and paternal DNA samples were extracted from parental blood samples obtained at the study visit (when the women were 28 weeks pregnant), and offspring DNA was obtained from cord blood at birth.

Genotyping of 2768 EFSOCH samples (n=969 mothers, 937 fathers and 862 children) was performed using the Illumina Infinium HumanCoreExome-24 array (n=551,839 SNPs/indels). Individual DNA samples with genotype call rate <98% were removed (n=50 individuals [1.8%]). SNPs were removed if they had call rates <95% (n=13,151 SNPs), showed evidence of deviation from Hardy-Weinberg equilibrium ($P<1x10^{-6}$; n=455 further SNPs), or had a minor allele frequency (MAF) <1% (n=257,289 further SNPs). Genotypically-derived sex information was compared with sex information in the phenotype file, and mismatched samples were excluded (n=13 individuals [0.61%]). Kinship was estimated using King [48]. Where evidence of labelling errors was clear, labels were updated, otherwise samples with kinship errors were excluded (n=22 individuals [0.79%]). Principal component analysis was performed to assess ancestry of the sample using flashPCA [49]. Outliers were defined as >4.56 SD from the cluster mean (defined using 1000 Genomes European data as the reference) and excluded (n=21 individuals [0.76%]). Imputation was performed using the Michigan imputation server and samples were imputed to the Haplotype Reference Consortium HRC v1.1 reference panel. SNPs were included in the analysis if imputation quality score was >0.4 and MAF ≥1%. After imputation, and once all exclusions had been made, 7,674,771 SNPs were available for analysis in 2662 individuals (939 mothers, 911 fathers and 812 children). Analyses were adjusted for the genotyping batch.

Ethical approval for the Exeter Family of Childhood Health was given by the North and East Devon (UK) Local Research Ethics Committee (approval number 1104), and informed consent was obtained from the parents of the newborns.

FIN

The Finnish dataset (FIN) was collected for a genetic study of spontaneous preterm birth [22,50]. Briefly, whole blood samples were collected from more than 1,600 mother/child pairs from the Helsinki (southern Finland) University Hospitals between 2004 and 2014.

All the studied samples are of Finnish descent. Crown-rump length at the first ultrasound screening between 10+ and 13 weeks was used to determine the gestational age. 2,962 blood samples from mothers and children were genotyped. After genotype quality control (QC) procedure and applying the phenotype-based inclusion/exclusion criteria, 1,322 mothers were selected and used in the analysis. The study was approved by the Ethics Committee of the Helsinki University Central Hospital. Written informed consent was given by all participants.

GPN

The Genomic and Proteomic Network for Preterm Birth Research (GPN) study [51]is a multicenter observational genome-wide association study (GWAS) designed to determine the genetic predisposition to idiopathic preterm birth. Phenotype data and genotype data from 743 spontaneous preterm births (20 to less than 34 weeks gestation), and 752 controls (39 to less than 42 weeks gestation) of diverse ethnic background (White, Hispanics, African Americans, and Others) were collected. For this current study, we identified 419 mothers of European descent using PCA from the data downloaded from dbGaP (phs000714.v1.p1). The GPN-PBR protocol was approved by the Investigational Review Boards at all institutions.

HAPO

All pregnant women at less than 32 weeks of gestation were eligible for enrollment in HAPO unless they met one of several exclusion criteria. All participants gave written informed consent, and an external data monitoring committee provided oversight. Study phenotype collection methods and inclusion and exclusion criteria have been published elsewhere [52,53]. Participants underwent a 75-g oral glucose tolerance test (OGTT) at ~28 weeks' gestation. Maternal DNA was taken from blood collected into an EDTA tube at 2 h during the OGTT, when phenotypes of interest were measured, including glucose, blood pressure, weight, and height. Glucose and C-peptide were measured in a central laboratory [52,53], and DNA was prepared using the automated Autopure LS from Gentra Systems. Submitted for genotyping were 3,152 European ancestry mother and offspring HAPO samples, of which 2,797 survived quality control (QC). DNA samples were genotyped using the Illumina Human 610 Quad v1 B SNP array at the Broad Institute following agreed-upon protocols of the Gene-Environment Association Studies (GENEVA) consortium [54]. Genotype data that passed initial QC at the genotyping centers were released to the GENEVA Coordinating Center (CC), National Center for Biotechnology Information database of Genotypes and Phenotypes (dbGaP), and HAPO study teams, who collectively performed QC using procedures previously described by the GENEVA consortium [54]. Poorly performing samples or SNPs were removed based on misspecified sex, chromosomal anomalies, unintended sample duplicates, sample relatedness, low call rate, high number of Mendelian errors, departures from Hardy-Weinberg equilibrium, duplicate discordance, sex differences in heterozygosity, and low minor allele frequencies as described elsewhere [54,55].

For the analysis of parental transmitted and non-transmitted alleles, we utilized phenotype and genotype data downloaded from dbGaP (phs000096.v2.p1).

HUNT

From 2012-2015 the HUNT-Michigan (HUNT-MI) collaboration genotyped approximately 72.000 individuals from the HUNT biobank [56]. The genotyping effort was a research collaboration between researchers at NTNU and the University of Michigan. Every individual with a DNA sample with a suitable DNA concentration was selected for genotyping. Samples were picked at random and genotyped in batches. All genotyping was performed at the Genomics-Core Facility (GCF) at the Norwegian University of Science and Technology, NTNU. The Trøndelag Health Study (HUNT) is a large population-based cohort from the county Trøndelag in Norway. All residents in the county, aged 20 years and older, have been invited to participate. Data was collected through three cross-sectional surveys, HUNT1 (1984-1986), HUNT2 (1995-1997) and HUNT3 (2006- 2008), and has been described in detail previously [57], with the fourth survey recently completed (HUNT4, 2017-2019). DNA from whole blood was collected from HUNT2 and HUNT3, with genotypes available from 71,860 participants. All genotyped participants have signed a written informed consent regarding the use of data from questionnaires, biological samples and linkage to other registries for research purposes.

In total, DNA from 71,860 HUNT samples was genotyped using one of three different Illumina HumanCoreExome arrays (HumanCoreExome12 v1.0, HumanCoreExome12 v1.1 and UM HUNT Biobank v1.0). Samples that failed to reach a 99% call rate, had contamination > 2.5% as estimated with BAF Regress [58], large chromosomal copy number variants, lower call rate of a technical duplicate pair and twins, gonosomal constellations other than XX and XY, or whose inferred sex contradicted the reported gender, were excluded. Samples that passed quality control were analysed in a second round of genotype calling following the Genome Studio quality control protocol described elsewhere [59]. Genomic position, strand orientation and the reference allele of genotyped variants were determined by aligning their probe sequences against the human genome (Genome Reference Consortium Human genome build 37 and revised Cambridge Reference Sequence of the human mitochondrial DNA;http://genome.ucsc.edu) using BLAT [60]. Variants were excluded if (1) their probe sequences could not be perfectly mapped to the reference genome, cluster separation was < 0.3, Gentrain score was < 0.15, showed deviations from Hardy Weinberg equilibrium in unrelated samples of European ancestry with p-value < 0.0001), their call rate was < 99%, or another assay with higher call rate genotyped the same variant.

Ancestry of all samples was inferred by projecting all genotyped samples into the space of the principal components of the Human Genome Diversity Project (HGDP)

reference panel (938 unrelated individuals; downloaded from http://csg.sph.umich.edu/chaolong/LASER/) [61,62], using PLINK v1.90 [63]. Recent European ancestry was defined as samples that fell into an ellipsoid spanning exclusively European populations of the HGDP panel. The different arrays were harmonized by reducing to a set of overlapping variants and excluding variants that showed frequency differences > 15% between data sets, or that were monomorphic in one and had MAF > 1% in another data set. The resulting genotype data were phased using Eagle2 v2.3 [6] . If you need information about ancestry or population structure (principal components) please contact the K.G. Jebsen center for genetic epidemiology.
Imputation was performed on the 69,716 samples of recent European ancestry using Minimac3 (v2.0.1,http://genome.sph.umich.edu/wiki/Minimac3) [44] with default settings (2.5 Mb reference-based chunking with 500kb windows) and a customized Haplotype Reference consortium release 1.1 (HRC v1.1) for autosomal variants and HRC v1.1 for chromosome X variants [43] . The customized reference panel represented the merged panel of two reciprocally imputed reference panels: (1) 2,201 low-coverage whole-genome sequence samples from the HUNT study and (2) HRC v1.1 with 1,023 HUNT WGS samples removed before merging. We excluded imputed variants with Rsq < 0.3 resulting in over 24.9 million well-imputed variants. The study was approved by The Regional Committee for Medical Research Ethics (#2016/551).

Northern Finland Birth Cohort 1966

The Northern Finland Birth Cohort 1966 is a prospective follow-up study of children from the two northernmost provinces of Finland born in 1966 [64]. All individuals still living in northern Finland or the Helsinki area (n = 8,463) were contacted and invited for clinical examination. A total of 6007 participants attended the clinical examination at the participants' age of 31 years. DNA was extracted from blood samples given at the clinical examination (5,753 samples available) [65]. The subset with DNA is representative of the original cohort in terms of major environmental and social factors. Informed consent was obtained from all subjects. After performing standard sample QC we included 5,402 NFBC66 participants that were genotyped on an Illumina HumanCNV370DUO Analysis BeadChip. 329,401 variants were included in the imputation scaffold. Variants were imputed to the HRC reference r1.1 2016 [43] on the Michigan Imputation Server. Haplotypes were pre phased using ShapeIT v2 r790 [5] and imputation was performed using MACH. Prior to analysis we excluded variants monomorphic in this dataset. We used rvtests [66] for association testing. To correct for distant genetic relatedness and population stratification within NFBC1966 we calculated a genetic relationship matrix based on all SNPs with MAF > 5%.

NFBC data is available from the University of Oulu, Infrastructure for Population Studies. Permission to use the data can be applied for research purposes via electronic material request portal. In the use of data, we follow the EU general data protection regulation

(679/2016) and Finnish Data Protection Act. The use of personal data is based on cohort participant's written informed consent at his/her latest follow-up study, which may cause limitations to its use. Please, contact NFBC project center (NFBCprojectcenter@oulu.fi) and visit the cohort website (www.oulu.fi/nfbc) for more information.

Project Viva

Project Viva is a longitudinal pre-birth cohort established to examine the effects of events during early development on lifetime health outcomes [67]. Between 1999 and 2002, the study recruited women in early pregnancy from eight obstetric offices of Atrius Harvard Vanguard Medical Associates, a multispecialty group practice in eastern Massachusetts, US. Exclusion criteria included multiple gestation, inability to answer questions in English, gestational age ≥22 weeks at recruitment, and plans to move away from the study area before delivery. Of 2312 eligible women, 2100 were still enrolled at delivery and had a live birth. For this analysis, we restricted to 604 white participants with genetic data and excluded 19 with pre-eclampsia, 107 who received any fertility treatment, 14 with low blood pressure, 19 with gestational diabetes, 6 who smoked ≥10 cigarettes/day and 8 who drank >1 servings/day of alcohol, leaving 431 participants in the analysis sample.

Women reported the date of last menstrual period (LMP) at study enrollment (median 9.9 weeks gestation). We obtained the date of delivery from the hospital medical record. We calculated the length of gestation in days by subtracting the date of the LMP from the date of delivery. If gestational age according to the second trimester ultrasound differed from that according to the LMP by more than 10 days, we used the ultrasound result to determine gestational duration.

We did not include principal components as covariates because none of the first 10 principal components were associated with gestational age.

Genotyping, phasing, and imputation: Samples were genotyped using the Illumina Infinium Core Exome-24 array. For quality control, we removed duplicates and low-quality samples, samples with SNP call rate <95% and samples with sex mismatch. We filtered out SNPs with call rate <98% (~5K), monomorphic SNPs (~800K) and SNPs with HWE p <1e-8 (~10K). Phasing and imputation was completed on the Michigan Imputation Server using ShapeIT v2.r790 and reference panel 1000G Phase 3 v5.

All mothers provided written informed consent. Institutional review boards at all participating institutions gave approval for this study.

STORK

The STORK study is a prospective cohort of 1031 healthy pregnant women of Scandinavian heritage who registered for obstetric care at the Oslo University Hospital Rikshospitalet from 2001 to 2008 [68]. Exclusion criteria were multiple pregnancies, known history of type 1 or type 2 diabetes mellitus, and severe chronic diseases (pulmonary, cardiac, gastrointestinal or renal).

DNA samples were genotyped on the Illumina Infinium CoreExome chip using Illumina iScan by the Department of Clinical Sciences, Clinical Research Centre, Lund University, Malmö, Sweden. Of the 1031 individuals who provided blood samples, 529 participants had usable SNP data after genotyping and quality control described elsewhere [69]. All 529 women had data on gestational length.

The study was approved by the Regional Committee for Medical Research Ethics, Southern Norway, Oslo, Norway (reference number S-2014/224-0119a and S-07392a) and performed according to the Declaration of Helsinki. All participating women provided written informed consent.

STORK Groruddalen

The STORK Groruddalen study (STORK-G) is a population-based cohort which included 823 healthy women attending three public mother–child health clinics for antenatal care in the multi-ethnic area of Groruddalen, Oslo, Norway [70]. Women were eligible if they: (i) lived in the study districts; (ii) planned to give birth at one of two study hospitals; (iii) were <20 weeks of pregnant; (iv) could communicate in Norwegian or any of the eight translated languages; (v) were able to give an informed consent. Women with pregestational diabetes or in need of intensive hospital follow-up during pregnancy were excluded. The participation rate was 74%, varying from 63.9% to 82.6% across ethnic groups.

DNA samples were genotyped on the Illumina Infinium CoreExome chip using Illumina iScan by the Department of Clinical Sciences, Clinical Research Centre, Lund University, Malmö, Sweden. Of the 664 genotyped samples those with low call rate (i.e. < 95%, n=0), extreme heterozygosity (> |mean± (3xSD)|, n=1), mismatched gender (n=24) or cryptic relatedness (i.e. one individual (chosen at random) from each related pair, defined as genome-wide IBD > 0.185 (n=6) were excluded from analyses. Genetic ethnic origin was defined by ancestry informative principal component analysis based on the variance-standardized relationship matrix generated in PLINK 1.9 software package [63] were used. In STORK Groruddalen a total of 310 women with European ancestry are

present after quality control has been performed. 310 women of European ancestry had data on gestational length.

The study was approved by the Regional Committee for Medical Research Ethics, Southern Norway, Oslo, Norway (ref.number 2015/1035) and performed according to the Declaration of Helsinki. We obtained written informed consent from all participants before any study-related procedure.

The Genetics of Glucose regulation in Gestation and Growth (Gen3G)
Gen3G is a prospective population-based pre-birth cohort that recruited pregnant women receiving prenatal care at the Centre Hospitalier Universitaire de Sherbrooke (CHUS) in QC, Canada between January 2010 to June 2013 [71]. Participating women were enrolled in the first trimester of pregnancy. Exclusion criteria included history of overt diabetes or laboratory evidence of overt diabetes at the first trimester study visit (hemoglobin A1C ≥6.5% or glucose ≥185 mg/dl after a 50-gram glucose load), multiple pregnancy, and use of medications that affect glucose metabolism. Of 1024 eligible women, 873 were still enrolled at delivery and had a live birth. For this analysis, we restricted to 582 participants with genetic data and excluded 37 with pre-eclampsia or GDM or hypertension, 46 with a repeat c-section, 6 with breech presentation, 19 who smoked ≥10 cigarettes/day and 15 who were not of european ethnicity, leaving 459 participants in the analysis sample.

Women reported the date of last menstrual period (LMP) at study enrollment (median 9.6 weeks gestation). We obtained the date of delivery from the hospital medical record. We calculated the length of gestation in days by subtracting the date of the LMP from the date of delivery. If gestational age according to the second trimester ultrasound differed from that according to the LMP by more than 5 days, we used the ultrasound result to determine gestational duration.

Three principle components were included as covariates because they were associated with gestational age ($p<0.1$).

We isolated DNA from maternal blood buffy coats using the Gentra Puregene Blood Kit (Qiagen, Mississauga, ON, Canada). 598 women samples were genotyped using Illumina MEGAex arrays. For quality control, we removed duplicates and samples with SNP call rate <98%. We also filtered SNPs with call rate <95% (~141K), monomorphic SNPs and SNPs with HWE p <1e-8 (~757K) and SNPs with concordance between duplicates <90% (37). Before running the imputation, we also removed insertions/deletions (738), SNPs on chromosome XY and 0 (~9K), SNPs with minor allele frequency <0.01 (~269K) and duplicate SNPs with the lower call rate (~19K), After QC, we had data available on 582

women over 838,884 SNPs. We performed phasing and imputation on the Michigan Imputation Server using ShapeIT v2.r790 and reference panel 1000G Phase 3 v5.

Ethical approval was obtained from the CHUS ethic committee board and every participant gave written informed consent before enrolment in the study.

The Norwegian Mother, Father and Child Cohort Study

Study population

The Norwegian Mother, Father and Child Cohort Study (MoBa) is an open-ended cohort study that recruited pregnant women in Norway from 1999 to 2008. Approximately 114,500 children, 95,200 mothers, and 75,000 fathers of predominantly Norwegian ancestry were enrolled in the study from 50 hospitals all across Norway [72,73]. The project Better Health by Harvesting Biobanks (HARVEST) randomly selected 11,490 umbilical cord blood DNA samples from the biobank of this study for family triad genotyping, excluding samples matching any of the following criteria: (1) stillborn, (2) deceased, (3) twins, (4) non-existing data at the Norwegian Medical Birth Registry, (5) missing anthropometric measurements at birth in Medical Birth Registry, (6) pregnancies where the mother did not answer the first questionnaire (as a proxy for higher dropout rate), and (7) missing parental DNA samples. In 2016, HARVEST randomly selected a second set of 8,900 triads using the same criteria. The same year NORMENT selected 5,910 triads with the same selection criteria as HARVEST, and extended this with 3,209 triads in 2018.

Genotyping, phasing and imputation

Genotyping of the samples was performed in seven different batches on different Illumina platforms (HumanCoreExome-12 v.1.1 and HumanCoreExome-24 v.1.0, Illumina's Global Screening Array v.1.0, InfiniumOmniExpress-24v1.2 and HumanOmniExpress-24-v1.0). The Genome Reference Consortium Human Build 37 (GRCh37) reference genome was used for all annotations.

Genotypes were called in Illumina GenomeStudio (v.2011.1 and v.2.0.3). Cluster positions were identified from samples with call rate ≥ 0.98 and GenCall score ≥ 0.15. We excluded variants with low call rates, signal intensity, quality scores, and deviation from Hardy-Weinberg equilibrium (HWE) based on the following QC parameters: call rate < 98 %, cluster separation < 0.4, 10% GC-score < 0.3, AA T Dev > 0.025, HWE *p*-value $< 1 \times 10^{-6}$. Samples were excluded based on call rate < 98 % and heterozygosity excess > 4 SD. Study participants with recent white Nordic ancestry were included after merging with ancestry reference samples from the HapMap project (ver. 3).

Pre-phasing was conducted locally using Shapeit v2.790[5]. Imputation was performed at the Sanger Imputation Server with positional Burrows-Wheeler transform and HRC version 1.1 as reference panel [43].

Phenotype

The MoBa cohort is linked to the Medical Birth Registry of Norway, from where we extracted information on gestational duration, and medically initiated delivery (inductions or c-sections).Maternal health information prior to and during pregnancy, including gestational duration and parity, as well as complications of pregnancy and birth were recovered from the Medical Birth Registry of Norway. We defined spontaneous onset of delivery as a delivery initiated by spontaneous contractions or rupture of membranes. Deliveries initiated by induction methods, including the use of prostaglandins, oxytocin, amniotomy or any other induction procedure, or planned cesarean section were excluded for gestational duration, and whenever these occurred in preterm or post-term cases.

Association analysis

Prior to analysis, we used a greedy algorithm to exclude one individual from each set of related subjects, where sets were defined as two individuals with a kinship coefficient >0.125. All analyses were performed using BOLT-LMM [33] (gestational duration) or REGENIE [74] (preterm and post-term deliveries), using genotype dosage as input and including parity and the 10 first principal components as covariates.

Informed consent was obtained from all study participants. The administrative board of the Norwegian Mother, Father and Child Cohort Study led by the Norwegian Institute of Public Health approved the study protocol. The MoBa cohort is currently regulated by the Norwegian Health Registry Act. The study was approved by The Regional Ethics Committee for Medical Research Ethics South East Norway (#2015/2425).

The Preterm birth Genome Project

The Preterm birth Genome Project (PGP) is a consortium initiated by the Preterm Birth International Collaborative (PREBIC), the World Health Organisation (WHO), and the March of Dimes [75]. Participants in the PGPII and PGPIII cohorts were recruited between the years 2007 and 2010, and between years 2013 and 2016, respectively. For both cohorts, DNA samples and detailed phenotypic data were collected (based on the Optimal PREBIC dataset [76]) from women who delivered in Perth, Western Australia. Informed written consent was obtained from the study participants. Genetic samples were collected from peripheral blood or saliva on days one to three post-delivery. DNA

were extracted and genotyped separately for each cohort. Cleaned genotyped data were phased using Eagle2 [6] and imputed using Minimac3 and Minimac4 [44] against the Haplotype Reference Consortium (version r1.1 2016) reference panel [43] on the Michigan Imputation Server. Ethics approvals for PGPII and PGPIII were obtained from the Women's and Newborn Health Service (HREC approvals 1572EW and 2013030EW).

Cohort acknowledgements

23andMe
We would like to thank the research participants and employees of 23andMe for making this work possible.

ALSPAC
Core funding for ALSPAC is provided by the UK Medical Research Council and Wellcome (217065/Z/19/Z) and the University of Bristol. Genotyping of the ALSPAC maternal samples was funded by Wellcome (WT088806) and the offspring samples were genotyped by Sample Logistics and Genotyping Facilities at the Wellcome Sanger Institute and LabCorp (Laboratory Corporation of America) using support from 23andMe. A comprehensive list of grants funding is available on the ALSPAC website (http://www.bristol.ac.uk/alspac/external/documents/grant-acknowledgements.pdf). We are extremely grateful to all the families who took part in ALSPAC, the midwives for their help in recruiting them, and the whole ALSPAC team, which includes interviewers, computer and laboratory technicians, clerical workers, research scientists, volunteers, managers, receptionists and nurses.

BiB
Born in Bradford (BiB) data used in this research was funded by Wellcome (WT101597MA), a joint grant from the UK Medical Research Council (MRC) and UK Economic and Social Science Research Council (ESRC) (MR/N024397/1), the British Heart Foundation (CS/16/4/32482) and the National Institute for Health Research (NIHR) under its Collaboration for Applied Health Research and Care (CLAHRC) for Yorkshire and Humber and the Clinical Research Network (CRN). Born in Bradford is only possible because of the enthusiasm and commitment of the Children and Parents in BiB. We are grateful to all the participants, teachers, school staff, health professionals and researchers who have made Born in Bradford happen.

CHOP
The authors thank the network of primary care clinicians and the patients and families for their contribution to this project and to clinical research facilitated by the Pediatric

Research Consortium (PeRC) at The Children's Hospital of Philadelphia. R. Chiavacci, E. Dabaghyan, A. (Hope) Thomas, K. Harden, A. Hill, C. Johnson-Honesty, C. Drummond, S. Harrison, F. Salley, C. Gibbons, K. Lilliston, C. Kim, E. Frackelton, G. Otieno, K. Thomas, C. Hou, K. Thomas and M.L. Garris provided expert assistance with genotyping and/or data collection and management. The authors would also like to thank S. Kristinsson, L.A. Hermannsson and A. Krisbjšrnsson of Rafšrninn ehf for extensive software design and contributions.

DBDS
We thank deCODE genetics/Amgen, Bio- and Genomebank Denmark and Department of Clinical Immunology, Copenhagen University Hospital for financial support of this study.

DNBC
The Danish National Birth Cohort (DNBC) was established with a significant grant from the Danish National Research Foundation. Additional support was obtained from the Danish Regional Committees, the Pharmacy Foundation, the Egmont Foundation, the March of Dimes Birth Defects Foundation, the Health Foundation and other minor grants. The DNBC biobank is a part of the Danish National Biobank resource, which was established with the support of major grants from the Novo Nordisk Foundation, the Danish Medical Research Council and the Lundbeck Foundation.

The DNBC preterm delivery study (DNBC-PTD) is a nested study within the DNBC. The generation of GWAS genotype data for the DNBC-PTD sample was carried out within the Gene Environment Association Studies (GENEVA) consortium with funding provided through the National Institutes of Health's Genes, Environment, and Health Initiative (U01HG004423; U01HG004446; U01HG004438).

The Genomics of overweight in Young Adults (GOYA) study is a nested study within the DNBC, and conducted in collaboration with the MRC Integrative Epidemiology Unit at the University of Bristol (MC_UU_12013/1-9). The genotyping for DNBC-GOYA study was funded by the Wellcome Trust (WT 084762).

We thank dbGAP for depositing and hosting the phenotype and genotype data of the DNBC data set.

EFSOCH
The Exeter Family Study of Childhood Health (EFSOCH) was supported by South West NHS Research and Development, Exeter NHS Research and Development, the Darlington Trust and the Peninsula National Institute of Health Research (NIHR) Clinical Research Facility at the University of Exeter. The opinions given in this paper do not

necessarily represent those of NIHR, the NHS or the Department of Health. Genotyping of the EFSOCH study samples was funded by the Wellcome Trust and Royal Society grant 104150/Z/14/Z.

EGCUT

This study was funded by EU H2020 grant 692145, Estonian Research Council Grant IUT20-60, IUT24-6, and European Union through the European Regional Development Fund Project No. 2014-2020.4.01.15-0012 GENTRANSMED and 2014-2020.4.01.16-0125. Data analyzes were carried out in part in the High-Performance Computing Center of University of Tartu.

FIN

We thank the participants in the Finnish birth cohort as well as the research group who collected the data. We thank the infants and their parents who agreed to take part in the GPN study and the medical and nursing colleagues who collected that data. NIH (1R01HD101669-01A1), Burroughs Wellcome Fund (10172896), the March of Dimes Prematurity Research Center Ohio Collaborative, a grant from the Cincinnati Children's Hospital Medical Center (GAP/RIP).

FINNGEN

We want to acknowledge the participants and investigators of the FinnGen study.

Gen3G

The Gen3G prospective cohort was supported by the Fonds de Recherche du Québec – Santé (FRQS – subvention Fonctionnement – Recherche Clinique - grant #20697) and by a Canadian Institute of Health Research (CIHR) Operating grant (Institute of Nutrition, Metabolism and Diabetes; MOP- 115071).

Gen3G investigators would like to acknowledge all Gen3G participants, the research staff from the endocrinology group at the Centre de Recherche of the Centre Hospitalier Universitaire de Sherbrooke (CRCHUS), the clinical and research staff of the Clinique de Prélèvement en Grossesse du CHUS, and the clinical staff from the CHUS Obstetric Department. The CRCHUS is a FRQ-Sante affiliated research centre; recruitment and follow-up of Gen3G participants was possible based on the on-going support from the CRCHUS.

GPN

The GPN datasets used for the analyses described in this manuscript were obtained from dbGaP at https://www.ncbi.nlm.nih.gov/gap/ through dbGaP accession number

phs000714.v1.p1. We thank dbGAP for depositing and hosting the phenotype and genotype data of the GPN data set.

HAPO

This study was supported by National Institutes of Health (NIH) grants HD-34242, HD-34243, HG-004415, and CA-141688, and by the American Diabetes Association. The authors are indebted to the participants of the HAPO Study at the following centers: Newcastle and Brisbane, Australia; Toronto, Canada; and Belfast, U.K. We thank dbGAP for depositing and hosting the phenotype and genotype data of the HAPO data set.

HUNT

The Trøndelag Health Study (HUNT) is a collaboration between HUNT Research Centre (Faculty of Medicine and Health Sciences, Norwegian University of Science and Technology NTNU), Trøndelag County Council, Central Norway Regional Health Authority, and the Norwegian Institute of Public Health. The genetic investigations of the HUNT Study, is a collaboration between researchers from the K.G. Jebsen center for genetic epidemiology and University of Michigan Medical School and the University of Michigan School of Public Health. The K.G. Jebsen Center for Genetic Epidemiology is financed by Stiftelsen Kristian Gerhard Jebsen; Faculty of Medicine and Health Sciences, NTNU, Norway.

MoBa

This work was supported by grants (B.J.) from The Swedish Research Council, Stockholm, Sweden (2015-02559), The Research Council of Norway, Oslo, Norway (FRIMEDBIO #547711, #273291), March of Dimes (#21-FY16-121) and (to P.R.N.) from the European Research Council (AdG SELECTionPREDISPOSED #293574), the Bergen Research Foundation ("Utilizing the Mother and Child Cohort and the Medical Birth Registry for Better Health"), Stiftelsen Kristian Gerhard Jebsen (Translational Medical Center), the University of Bergen, the Research Council of Norway (FRIPRO grant #240413), the Western Norway Regional Health Authority (Strategic Fund "Personalized Medicine for Children and Adults"), the Novo Nordisk Foundation (grant #54741), and the Norwegian Diabetes Association. This work was partly supported by the Research Council of Norway through its Centres of Excellence funding scheme (#262700, #223273), Better Health by Harvesting Biobanks (#229624) and The Norwegian Mother, Father and Child Cohort Study is supported by the Norwegian Ministry of Health and Care Services and the Ministry of Education and Research, NIH/NIEHS (contract no N01-ES-75558), NIH/NINDS (grant no.1 UO1 NS 047537-01 and grant no.2 UO1 NS 047537-06A1). We are grateful to all the families in Norway who are taking part in this ongoing cohort study. All analyses were performed using digital labs in HUNT Cloud at the Norwegian University of Science and Technology, Trondheim, Norway.

The Norwegian Mother, Father and Child Cohort Study is supported by the Norwegian Ministry of Health and Care Services and the Ministry of Education and Research. We are grateful to all the participating families in Norway who take part in this on-going cohort study. We thank the Norwegian Institute of Public Health (NIPH) for generating high-quality genomic data. We thank the Norwegian Institute of Public Health (NIPH) for generating high-quality genomic data. This research is part of the HARVEST collaboration, supported by the Research Council of Norway (#229624). We also thank the NORMENT Centre for providing genotype data, funded by the Research Council of Norway (#223273), South East Norway Health Authority and KG Jebsen Stiftelsen. We further thank the Center for Diabetes Research, the University of Bergen for providing genotype data and performing quality control and imputation of the data funded by the ERC AdG project SELECTionPREDISPOSED, Stiftelsen Kristian Gerhard Jebsen, Trond Mohn Foundation, the Research Council of Norway, the Novo Nordisk Foundation, the University of Bergen, and the Western Norway health Authorities (Helse Vest). All analyses were performed using digital labs in HUNT Cloud at the Norwegian University of Science and Technology, Trondheim, Norway.

NFBC

NFBC1966 received financial support from the Academy of Finland (project grants 104781, 120315, 129269, 1114194, 24300796, Center of Excellence in Complex Disease Genetics and SALVE), University Hospital Oulu, Biocenter, University of Oulu, Finland (75617), NHLBI grant 5R01HL087679-02 through the STAMPEDE program (1RL1MH083268-01), NIH/NIMH (5R01MH63706:02), ENGAGE project and grant agreement HEALTH-F4-2007-201413, EU FP7 EurHEALTHAgeing -277849, the Medical Research Council, UK (G0500539, G0600705, G1002319, PrevMetSyn/SALVE) and the MRC, Centenary Early Career Award. The program is currently being funded by the H2020-633595 DynaHEALTH action, academy of Finland EGEA-project (285547) and EU H2020 ALEC project (Grant Agreement 633212).

PGP

This study was funded by the World Health Organisation, March of Dimes, National Health & Medical Research Council (APP1042267).

Project Viva

Project Viva was supported by grants from the US National Institutes of Health (UH3 OD23286 and R01 HD034568). We thank the participants and staff of Project Viva.

STORK-G
The authors thank study staff and general staff at the Child Health Clinics in Stovner, Grorud and Bjerke districts in Oslo and at the delivery- and post-natal wards at Akershus University Hospital and Oslo University Hospital, for help with collecting the data, and the Hormone Laboratory, Oslo University Hospital for DNA-extraction.

STORK
The STORK study received additional funding from the Norwegian Diabetes Association, the Norwegian Odd Fellow Research Fund and Johan Selmer Kvanes' Endowment for Research in Diabetes.

UK-Biobank

We want to thank the participants of the UK Biobank and the investigators of the Pan-UK Biobank team.

WTCCC58BC
The management of the 1958 Birth Cohort is funded by the Economic and Social Research Council (grant number ES/M001660/1). Access to these resources was enabled via the 58READIE Project funded by Wellcome Trust and Medical Research Council (grant numbers WT095219MA and G1001799). DNA collection was funded by MRC grant G0000934 and cell-line creation by Wellcome Trust grant 068545/Z/02. This study makes use of data generated by the Wellcome Trust Case-Control Consortium. A full list of investigators who contributed to generation of the data is available from the Wellcome Trust Case-Control Consortium website. Funding for the project was provided by the Wellcome Trust under the award 076113. This research used resources provided by the Type 1 Diabetes Genetics Consortium, a collaborative clinical study sponsored by the National Institute of Diabetes and Digestive and Kidney Diseases (NIDDK), National Institute of Allergy and Infectious Diseases, National Human Genome Research Institute, National Institute of Child Health and Human Development, and Juvenile Diabetes Research Foundation International (JDRF) and supported by U01 DK062418.

Bibliography

1. Pique-Regi, R. *et al.* A single-cell atlas of the myometrium in human parturition. *JCI Insight* **7**, e153921 (2022).
2. Juliusdottir, T. *et al.* Distinction between the effects of parental and fetal genomes on fetal growth. *Nat. Genet.* **53**, 1135–1142 (2021).

3. Kong, A. *et al.* Detection of sharing by descent, long-range phasing and haplotype imputation. *Nat. Genet.* **40**, 1068–1075 (2008).
4. Kong, A. *et al.* Parental origin of sequence variants associated with complex diseases. *Nature* **462**, 868–874 (2009).
5. O'Connell, J. *et al.* A general approach for haplotype phasing across the full spectrum of relatedness. *PLoS Genet.* **10**, e1004234 (2014).
6. Loh, P.-R. *et al.* Reference-based phasing using the Haplotype Reference Consortium panel. *Nat. Genet.* **48**, 1443–1448 (2016).
7. Privé, F., Arbel, J. & Vilhjálmsson, B. J. LDpred2: better, faster, stronger. *Bioinformatics* **36**, 5424–5431 (2020).
8. Loh, P.-R., Kichaev, G., Gazal, S., Schoech, A. P. & Price, A. L. Mixed-model association for biobank-scale datasets. *Nat. Genet.* **50**, 906–908 (2018).
9. O'Connor, L. J. & Price, A. L. Distinguishing genetic correlation from causation across 52 diseases and complex traits. *Nat. Genet.* **50**, 1728–1734 (2018).
10. Zhu, Z. *et al.* Causal associations between risk factors and common diseases inferred from GWAS summary data. *Nat. Commun.* **9**, 224 (2018).
11. Warrington, N. M. *et al.* Maternal and fetal genetic effects on birth weight and their relevance to cardio-metabolic risk factors. *Nat. Genet.* **51**, 804–814 (2019).
12. Liu, X. *et al.* Variants in the fetal genome near pro-inflammatory cytokine genes on 2q13 associate with gestational duration. *Nat. Commun.* **10**, 3927 (2019).
13. Bulik-Sullivan, B. K. *et al.* LD Score regression distinguishes confounding from polygenicity in genome-wide association studies. *Nat. Genet.* **47**, 291–295 (2015).
14. Clogg, C. C., Petkova, E. & Haritou, A. Statistical methods for comparing regression coefficients between models. *Am. J. Sociol.* **100**, 1261–1293 (1995).
15. Generality of the basic inverse-variance method. in *Introduction to Meta-Analysis* 311–319 (John Wiley & Sons, Ltd, 2009). doi:10.1002/9780470743386.ch34.

16. Burgess, S. & Thompson, S. G. Interpreting findings from Mendelian randomization using the MR-Egger method. *Eur. J. Epidemiol.* **32**, 377–389 (2017).
17. Yavorska, O. O. & Burgess, S. MendelianRandomization: an R package for performing Mendelian randomization analyses using summarized data. *Int. J. Epidemiol.* **46**, 1734–1739 (2017).
18. Siewert, K. M. & Voight, B. F. Detecting long-term balancing selection Using allele frequency correlation. *Mol. Biol. Evol.* **34**, 2996–3005 (2017).
19. Md, R., Mj, H., I, G. & A, S. Genome-wide inference of ancestral recombination graphs. *PLoS Genet.* **10**, (2014).
20. Davydov, E. V. *et al.* Identifying a high fraction of the human genome to be under selective constraint using GERP++. *PLoS Comput. Biol.* **6**, e1001025 (2010).
21. Huang, Y.-F., Gulko, B. & Siepel, A. Fast, scalable prediction of deleterious noncoding variants from functional and population genomic data. *Nat. Genet.* **49**, 618–624 (2017).
22. Zhang, G. *et al.* Genetic Associations with Gestational Duration and Spontaneous Preterm Birth. *N. Engl. J. Med.* **377**, 1156–1167 (2017).
23. 1000 Genomes Project Consortium *et al.* A global reference for human genetic variation. *Nature* **526**, 68–74 (2015).
24. Boyd, A. *et al.* Cohort Profile: the 'children of the 90s'--the index offspring of the Avon Longitudinal Study of Parents and Children. *Int. J. Epidemiol.* **42**, 111–127 (2013).
25. Fraser, A. *et al.* Cohort Profile: the Avon Longitudinal Study of Parents and Children: ALSPAC mothers cohort. *Int. J. Epidemiol.* **42**, 97–110 (2013).

26. Wright, J. *et al.* Cohort Profile: the Born in Bradford multi-ethnic family cohort study. *Int. J. Epidemiol.* **42**, 978–991 (2013).
27. Power, C. & Elliott, J. Cohort profile: 1958 British birth cohort (National Child Development Study). *Int. J. Epidemiol.* **35**, 34–41 (2006).
28. International Multiple Sclerosis Genetics Consortium *et al.* Genetic risk and a primary role for cell-mediated immune mechanisms in multiple sclerosis. *Nature* **476**, 214–219 (2011).
29. Barrett, J. C. *et al.* Genome-wide association study and meta-analysis find that over 40 loci affect risk of type 1 diabetes. *Nat. Genet.* **41**, 703–707 (2009).
30. Hyppönen, E., Power, C. & Smith, G. D. Parental growth at different life stages and offspring birthweight: an intergenerational cohort study. *Paediatr. Perinat. Epidemiol.* **18**, 168–177 (2004).
31. Hansen, T. F. *et al.* DBDS Genomic Cohort, a prospective and comprehensive resource for integrative and temporal analysis of genetic, environmental and lifestyle factors affecting health of blood donors. *BMJ Open* **9**, e028401 (2019).
32. Bliddal, M., Broe, A., Pottegård, A., Olsen, J. & Langhoff-Roos, J. The Danish Medical Birth Register. *Eur. J. Epidemiol.* **33**, 27–36 (2018).
33. Loh, P.-R. *et al.* Efficient Bayesian mixed-model analysis increases association power in large cohorts. *Nat. Genet.* **47**, 284–290 (2015).
34. Zhou, W. *et al.* Efficiently controlling for case-control imbalance and sample relatedness in large-scale genetic association studies. *Nat. Genet.* **50**, 1335–1341 (2018).
35. Gudbjartsson, D. F. *et al.* Large-scale whole-genome sequencing of the Icelandic

population. *Nat. Genet.* **47**, 435–444 (2015).

36. Jónsson, H. *et al.* Whole genome characterization of sequence diversity of 15,220 Icelanders. *Sci. Data* **4**, 170115 (2017).

37. McKenna, A. *et al.* The genome analysis toolkit: a MapReduce framework for analyzing next-generation DNA sequencing data. *Genome Res.* **20**, 1297–1303 (2010).

38. Olsen, J. *et al.* The Danish National Birth Cohort--its background, structure and aim. *Scand. J. Public Health* **29**, 300–307 (2001).

39. Lynge, E., Sandegaard, J. L. & Rebolj, M. The Danish National Patient Register. *Scand. J. Public Health* **39**, 30–33 (2011).

40. Paternoster, L. *et al.* Genome-wide population-based association study of extremely overweight young adults--the GOYA study. *PloS One* **6**, e24303 (2011).

41. Ryckman, K. K. *et al.* Replication of a genome-wide association study of birth weight in preterm neonates. *J. Pediatr.* **160**, 19-24.e4 (2012).

42. Cornelis, M. C. *et al.* The Gene, Environment Association Studies consortium (GENEVA): maximizing the knowledge obtained from GWAS by collaboration across studies of multiple conditions. *Genet. Epidemiol.* **34**, 364–372 (2010).

43. McCarthy, S. *et al.* A reference panel of 64,976 haplotypes for genotype imputation. *Nat. Genet.* **48**, 1279–1283 (2016).

44. Das, S. *et al.* Next-generation genotype imputation service and methods. *Nat. Genet.* **48**, 1284–1287 (2016).

45. Marchini, J. & Howie, B. Genotype imputation for genome-wide association studies. *Nat. Rev. Genet.* **11**, 499–511 (2010).

46. Leitsalu, L. *et al.* Cohort Profile: Estonian Biobank of the Estonian Genome Center,

University of Tartu. *Int. J. Epidemiol.* **44**, 1137–1147 (2015).

47. Knight, B., Shields, B. M. & Hattersley, A. T. The Exeter Family Study of Childhood Health (EFSOCH): study protocol and methodology. *Paediatr. Perinat. Epidemiol.* **20**, 172–179 (2006).

48. Manichaikul, A. *et al.* Robust relationship inference in genome-wide association studies. *Bioinforma. Oxf. Engl.* **26**, 2867–2873 (2010).

49. Abraham, G. & Inouye, M. Fast principal component analysis of large-scale genome-wide data. *PloS One* **9**, e93766 (2014).

50. Plunkett, J. *et al.* An evolutionary genomic approach to identify genes involved in human birth timing. *PLoS Genet.* **7**, e1001365 (2011).

51. Zhang, H. *et al.* A genome-wide association study of early spontaneous preterm delivery. *Genet. Epidemiol.* **39**, 217–226 (2015).

52. HAPO Study Cooperative Research Group. The Hyperglycemia and Adverse Pregnancy Outcome (HAPO) Study. *Int. J. Gynaecol. Obstet. Off. Organ Int. Fed. Gynaecol. Obstet.* **78**, 69–77 (2002).

53. HAPO Study Cooperative Research Group *et al.* Hyperglycemia and adverse pregnancy outcomes. *N. Engl. J. Med.* **358**, 1991–2002 (2008).

54. Laurie, C. C. *et al.* Quality control and quality assurance in genotypic data for genome-wide association studies. *Genet. Epidemiol.* **34**, 591–602 (2010).

55. Hayes, M. G. *et al.* Identification of HKDC1 and BACE2 as genes influencing glycemic traits during pregnancy through genome-wide association studies. *Diabetes* **62**, 3282–3291 (2013).

56. Brumpton, B. M. *et al.* The HUNT study: A population-based cohort for genetic

research. *Cell Genomics* **2**, 100193 (2022).

57. Krokstad, S. *et al.* Cohort Profile: the HUNT Study, Norway. *Int. J. Epidemiol.* **42**, 968–977 (2013).
58. Jun, G. *et al.* Detecting and estimating contamination of human DNA samples in sequencing and array-based genotype data. *Am. J. Hum. Genet.* **91**, 839–848 (2012).
59. Guo, Y. *et al.* Illumina human exome genotyping array clustering and quality control. *Nat. Protoc.* **9**, 2643–2662 (2014).
60. ENCODE Project Consortium. An integrated encyclopedia of DNA elements in the human genome. *Nature* **489**, 57–74 (2012).
61. Li, J. Z. *et al.* Worldwide human relationships inferred from genome-wide patterns of variation. *Science* **319**, 1100–1104 (2008).
62. Wang, C. *et al.* Ancestry estimation and control of population stratification for sequence-based association studies. *Nat. Genet.* **46**, 409–415 (2014).
63. Chang, C. C. *et al.* Second-generation PLINK: rising to the challenge of larger and richer datasets. *GigaScience* **4**, 7 (2015).
64. Rantakallio, P. The longitudinal study of the northern Finland birth cohort of 1966. *Paediatr. Perinat. Epidemiol.* **2**, 59–88 (1988).
65. Sovio, U. *et al.* Genetic determinants of height growth assessed longitudinally from infancy to adulthood in the northern Finland birth cohort 1966. *PLoS Genet.* **5**, e1000409 (2009).
66. Zhan, X., Hu, Y., Li, B., Abecasis, G. R. & Liu, D. J. RVTESTS: an efficient and comprehensive tool for rare variant association analysis using sequence data. *Bioinforma. Oxf. Engl.* **32**, 1423–1426 (2016).

67. Oken, E. *et al.* Cohort profile: project viva. *Int. J. Epidemiol.* **44**, 37–48 (2015).
68. Voldner, N. Modifiable determinants of newborn macrosomia and birth complications. (2009).
69. Moen, G.-H. *et al.* Genetic determinants of glucose levels in pregnancy: genetic risk scores analysis and GWAS in the Norwegian STORK cohort. *Eur. J. Endocrinol.* **179**, 363–372 (2018).
70. Jenum, A. K. *et al.* The STORK Groruddalen research programme: A population-based cohort study of gestational diabetes, physical activity, and obesity in pregnancy in a multiethnic population. Rationale, methods, study population, and participation rates. *Scand. J. Public Health* **38**, 60–70 (2010).
71. Guillemette, L. *et al.* Genetics of Glucose regulation in Gestation and Growth (Gen3G): a prospective prebirth cohort of mother-child pairs in Sherbrooke, Canada. *BMJ Open* **6**, e010031 (2016).
72. Magnus, P. *et al.* Cohort Profile Update: The Norwegian Mother and Child Cohort Study (MoBa). *Int. J. Epidemiol.* **45**, 382–388 (2016).
73. Sole-Navais, P. *et al.* Autozygosity mapping and time-to-spontaneous delivery in Norwegian parent-offspring trios. *Hum. Mol. Genet.* (2020) doi:10.1093/hmg/ddaa255.
74. Mbatchou, J. *et al.* Computationally efficient whole-genome regression for quantitative and binary traits. *Nat. Genet.* **53**, 1097–1103 (2021).
75. Biggio, J. *et al.* A call for an international consortium on the genetics of preterm birth. *Am. J. Obstet. Gynecol.* **199**, 95–97 (2008).
76. Pennell, C. E. *et al.* Genetic epidemiologic studies of preterm birth: guidelines for

research. *Am. J. Obstet. Gynecol.* **196**, 107–118 (2007).

Supplementary figures

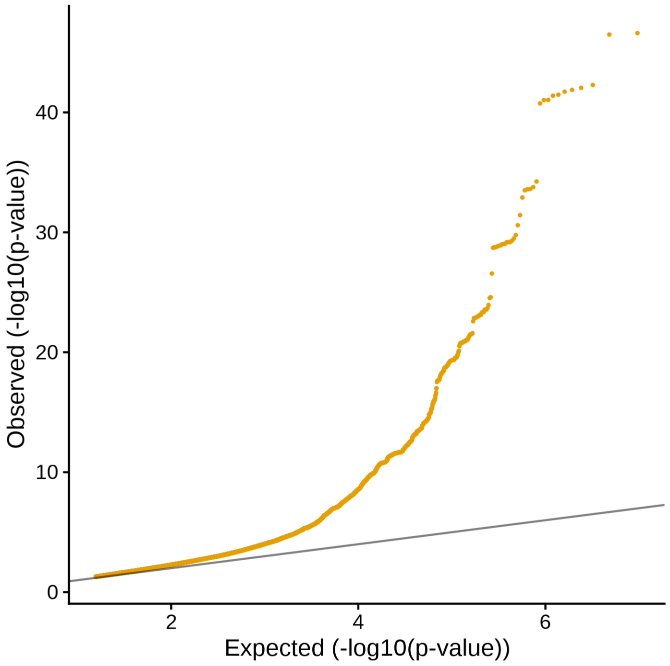

Supplementary Fig. 1. Quantile-quantile plot of gestational duration GWAS meta-analysis (n = 195,555). Observed p-values were two-sided, and were estimated using fixed effect inverse-variance weighted meta-analysis.

A

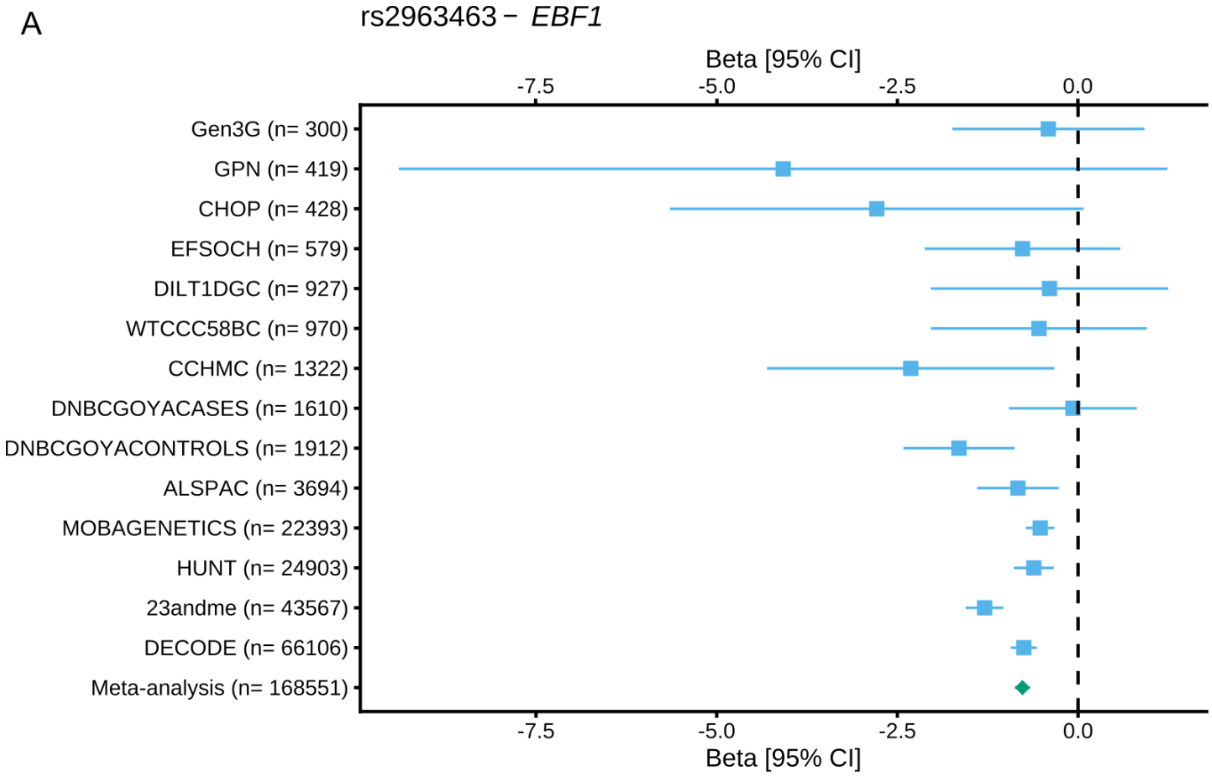
rs2963463 − EBF1
Beta [95% CI]
-7.5
-5.0
-2.5
0.0
Gen3G (n= 300)
GPN (n= 419)
CHOP (n= 428)
EFSOCH (n= 579)
DILT1DGC (n= 927)
WTCCC58BC (n= 970)
CCHMC (n= 1322)
DNBCGOYACASES (n= 1610)
DNBCGOYACONTROLS (n= 1912)
ALSPAC (n= 3694)
MOBAGENETICS (n= 22393)
HUNT (n= 24903)
23andme (n= 43567)
DECODE (n= 66106)
Meta-analysis (n= 168551)
-7.5
-5.0
-2.5
0.0
Beta [95% CI]

B

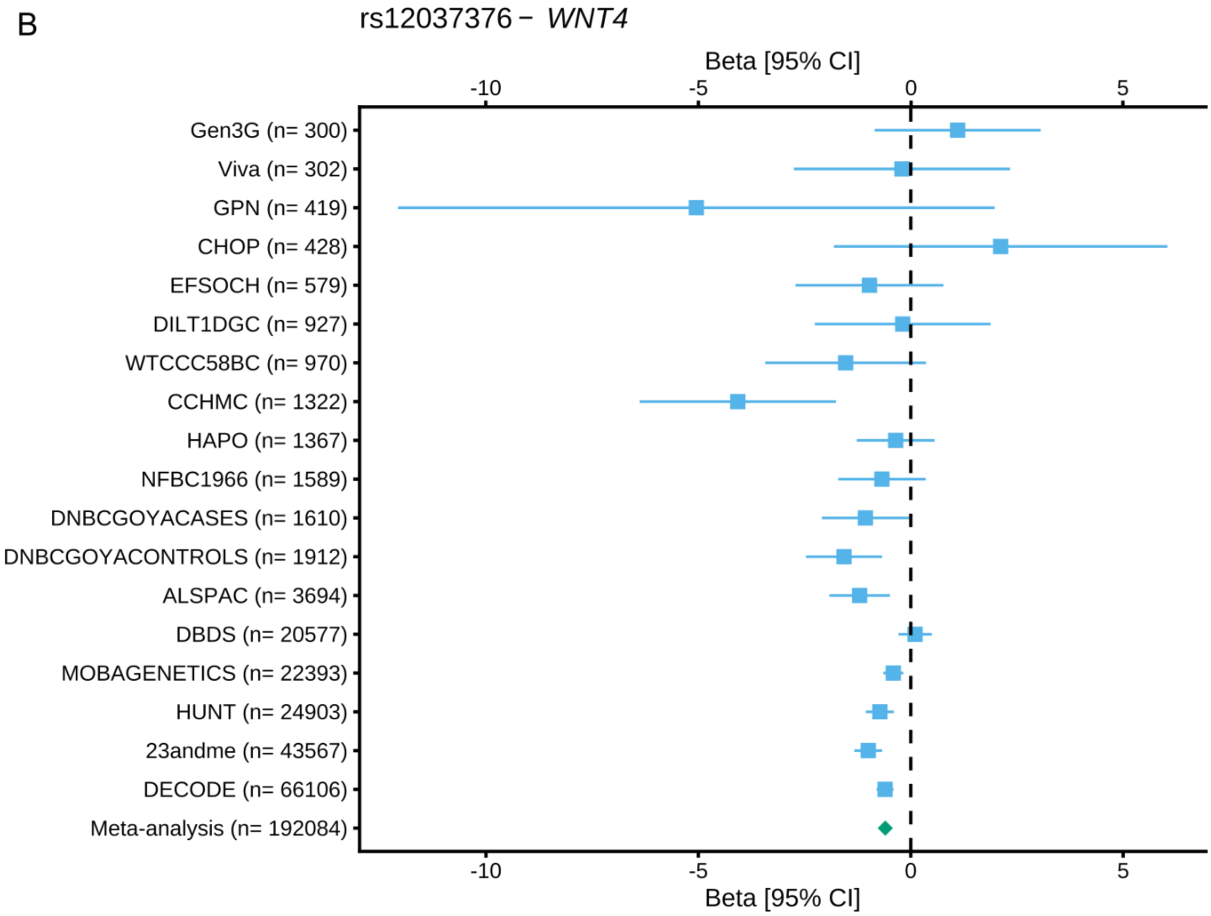
rs12037376 − WNT4
Beta [95% CI]
-10
-5
0
5
Gen3G (n= 300)
Viva (n= 302)
GPN (n= 419)
CHOP (n= 428)
EFSOCH (n= 579)
DILT1DGC (n= 927)
WTCCC58BC (n= 970)
CCHMC (n= 1322)
HAPO (n= 1367)
NFBC1966 (n= 1589)
DNBCGOYACASES (n= 1610)
DNBCGOYACONTROLS (n= 1912)
ALSPAC (n= 3694)
DBDS (n= 20577)
MOBAGENETICS (n= 22393)
HUNT (n= 24903)
23andme (n= 43567)
DECODE (n= 66106)
Meta-analysis (n= 192084)
-10
-5
0
5
Beta [95% CI]

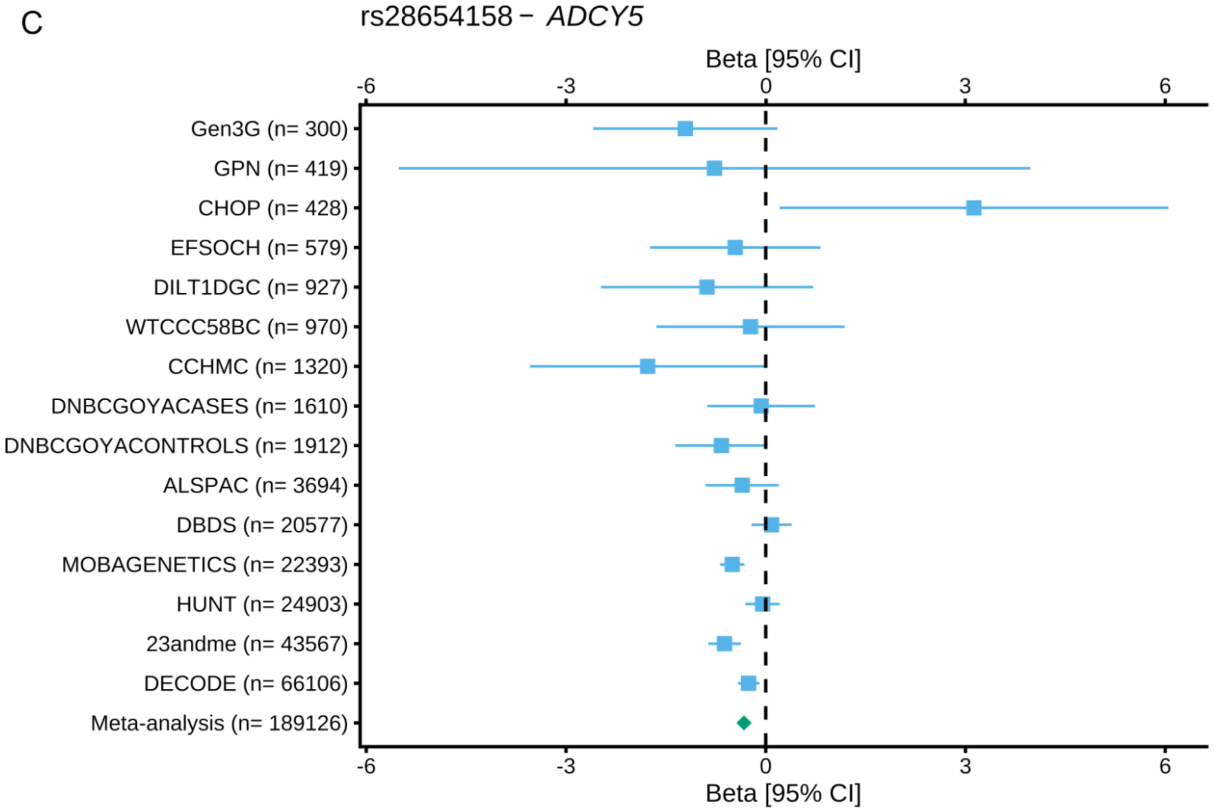
C
rs28654158 − *ADCY5*
Beta [95% CI]
-6
-3
0
3
6
Gen3G (n= 300)
GPN (n= 419)
CHOP (n= 428)
EFSOCH (n= 579)
DILT1DGC (n= 927)
WTCCC58BC (n= 970)
CCHMC (n= 1320)
DNBCGOYACASES (n= 1610)
DNBCGOYACONTROLS (n= 1912)
ALSPAC (n= 3694)
DBDS (n= 20577)
MOBAGENETICS (n= 22393)
HUNT (n= 24903)
23andme (n= 43567)
DECODE (n= 66106)
Meta-analysis (n= 189126)

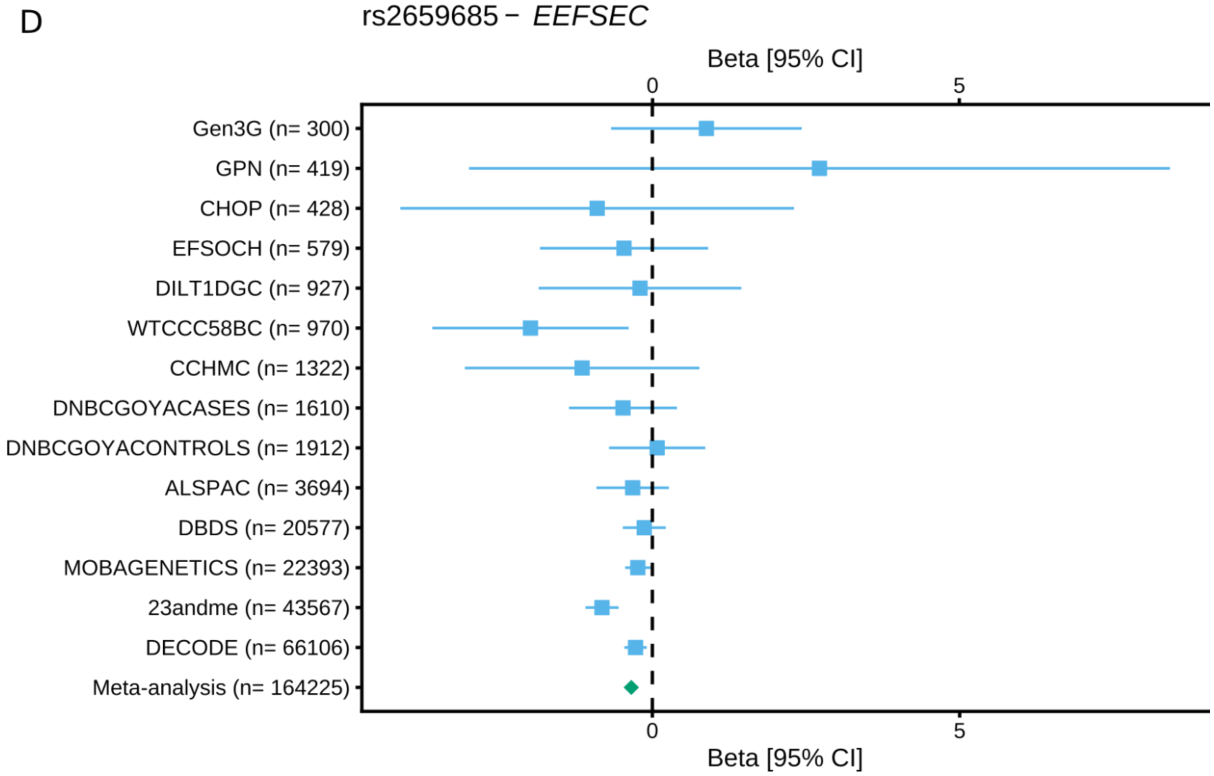
D
rs2659685 − *EEFSEC*
Beta [95% CI]
0
5
Gen3G (n= 300)
GPN (n= 419)
CHOP (n= 428)
EFSOCH (n= 579)
DILT1DGC (n= 927)
WTCCC58BC (n= 970)
CCHMC (n= 1322)
DNBCGOYACASES (n= 1610)
DNBCGOYACONTROLS (n= 1912)
ALSPAC (n= 3694)
DBDS (n= 20577)
MOBAGENETICS (n= 22393)
23andme (n= 43567)
DECODE (n= 66106)
Meta-analysis (n= 164225)

E

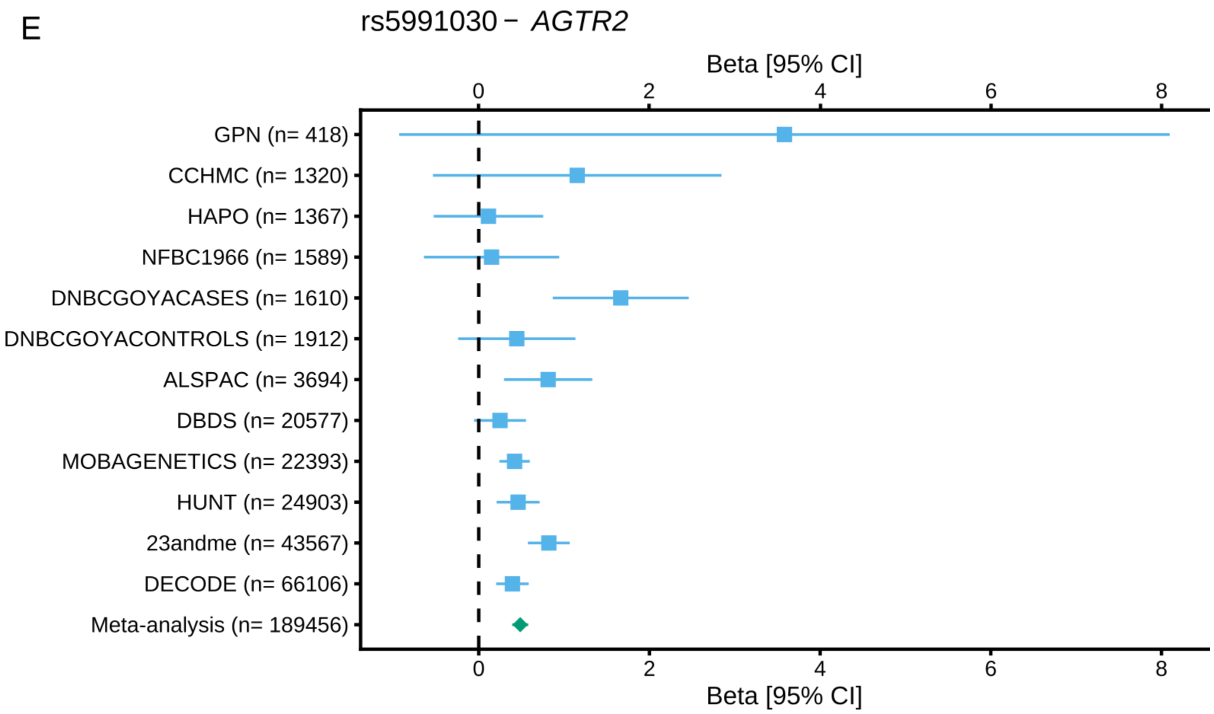

Supplementary Fig. 2. Forest plots of the five top variants associated with gestational duration that had significant heterogeneity after fixed effects inverse-variance weighted meta-analysis. Each square represents the effect size in days for a particular cohort and error bars are the 95% CI. Diamond represents the estimate after meta-analysis (also in days). The index variant for the following loci is shown: A) *EBF1*, B) *WNT4*, C) *EEFSEC*, D) *ADCY5* and E) *AGTR2*. Sample size for each study cohort and after meta-analysis is detailed in parentheses after the cohort name.

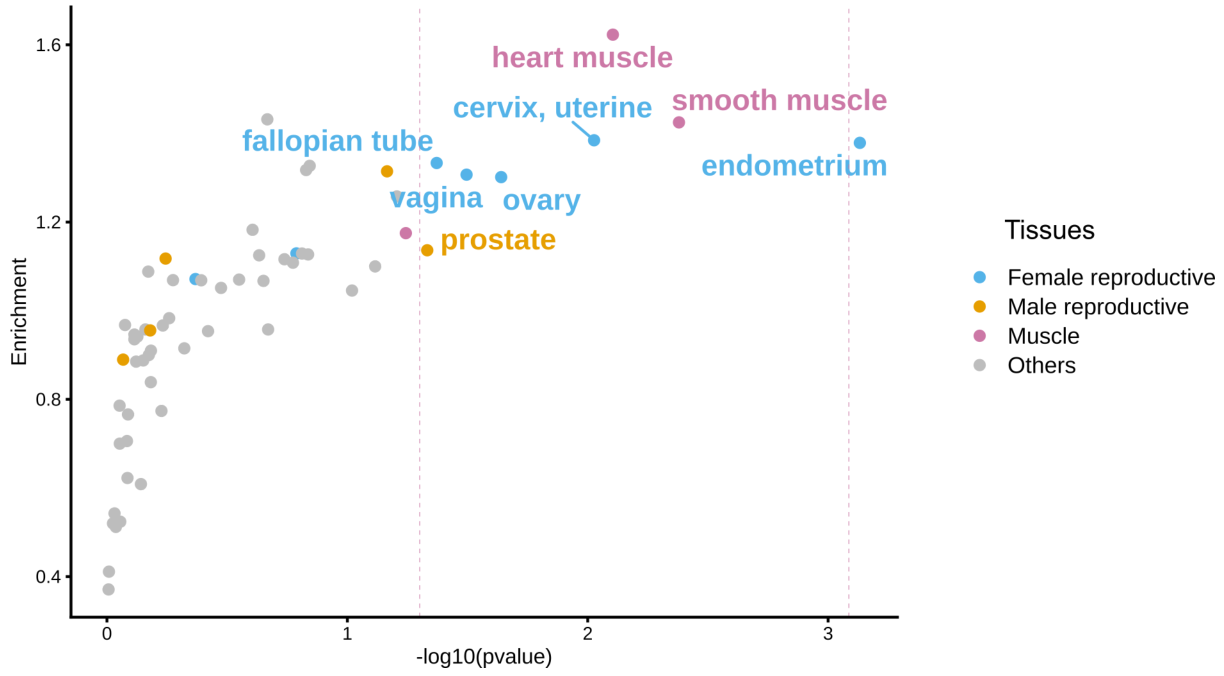

Supplementary Fig. 3. RNA tissue-specific enrichment of nearest protein coding genes to gestational duration index SNPs. Tissue-specific RNA was obtained from the Human Protein Atlas. The x-axis shows the -log10(p-value) for enrichment using a Wilcoxon test (two-sided), and the y-axis the enrichment (median in nearest protein coding genes / median all other genes). Vertical lines represent nominal significance (nearest to the y-axis) or significance threshold after Bonferroni correction for multiple comparisons (outermost vertical line, p-value = 0.05 / 65 tissues).

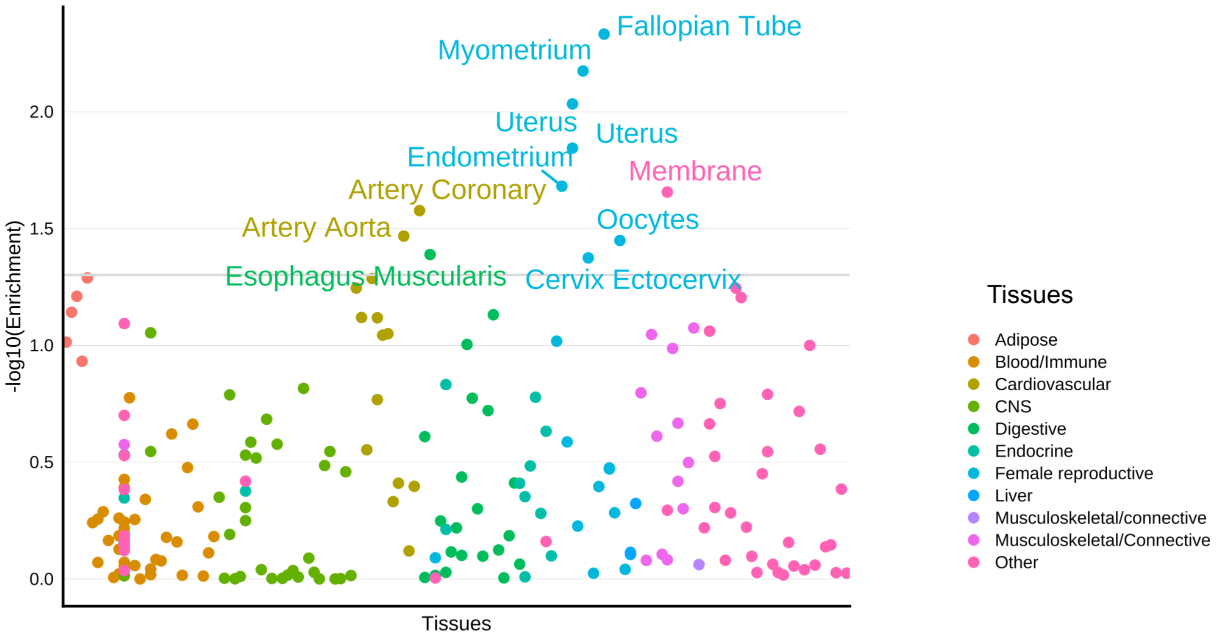

Supplementary Fig. 4. Enrichment of SNP-heritability of gestational duration for tissue-specific gene expression. Partitioned LD-score regression was used to estimate enrichment of SNP-heritability in 205 cell-types/ tissues, with pre-calculated LD-scores. Each dot represents a cell-type/ tissue; the ones that are labeled have a significant enrichment (two-sided p-value < 0.05, marked with a dark gray horizontal line). No adjustments for multiple comparisons were made.

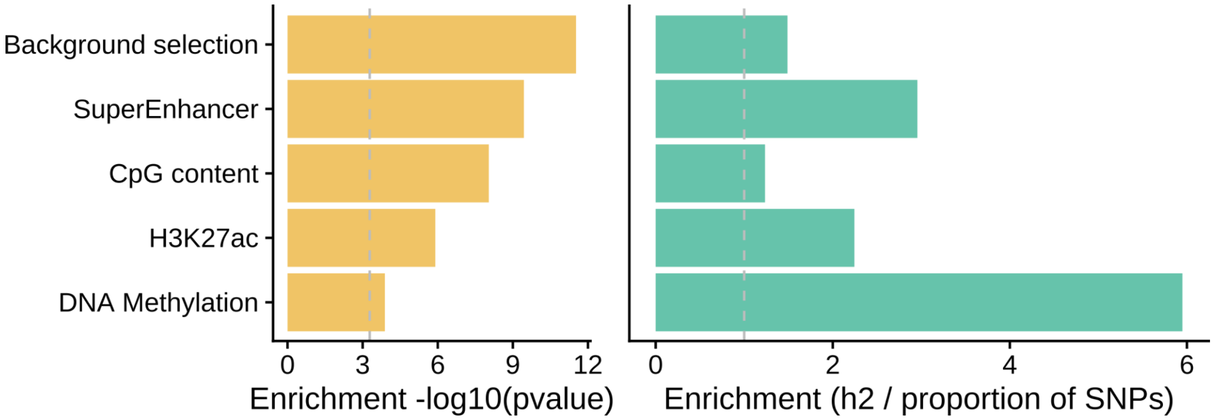

Supplementary Fig. 5. Enrichment p-values for partitioned LD-score regression analysis of gestational duration SNP heritability. The left plot shows the LD-score regression enrichment p-value for the five functional annotation categories that passed a Bonferroni correction for multiple comparisons (two-sided p-value< 0.05 / 97 categories).

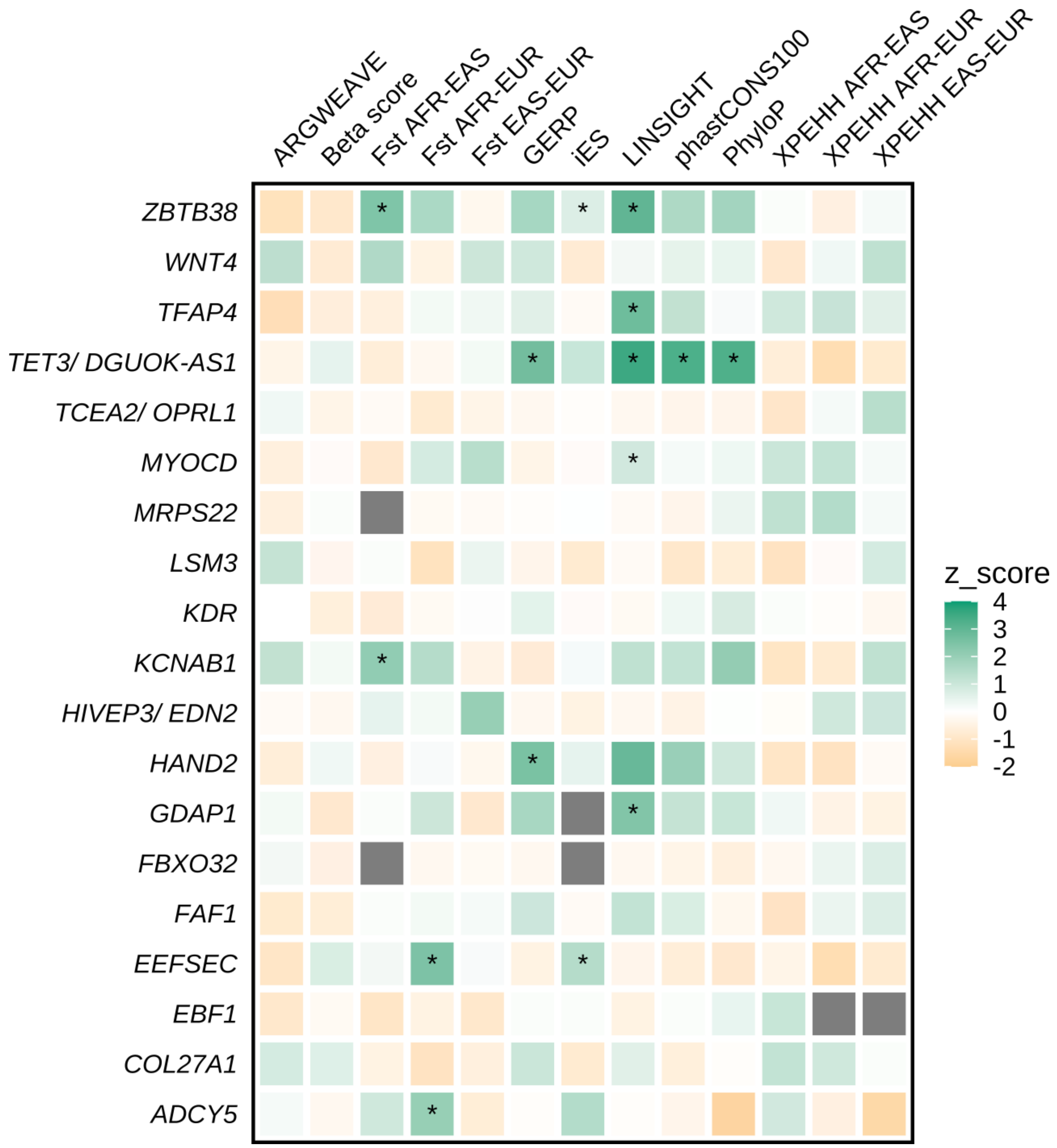

Supplementary Fig. 6. Evolutionary metrics enrichment for gestational duration loci. Enrichment analysis was performed using the MOSAIc pipeline for all regions with genome-wide significant associations with gestational duration, except the *HLA* gene region and the two in the X chromosome (*AGTR2* and *RAP2C*). The z-score for enrichment was obtained from a distribution that corresponds to the metric of the evolutionary force for 5,000 matched controls. The asterisk shows the regions significantly enriched for a specific evolutionary metric.

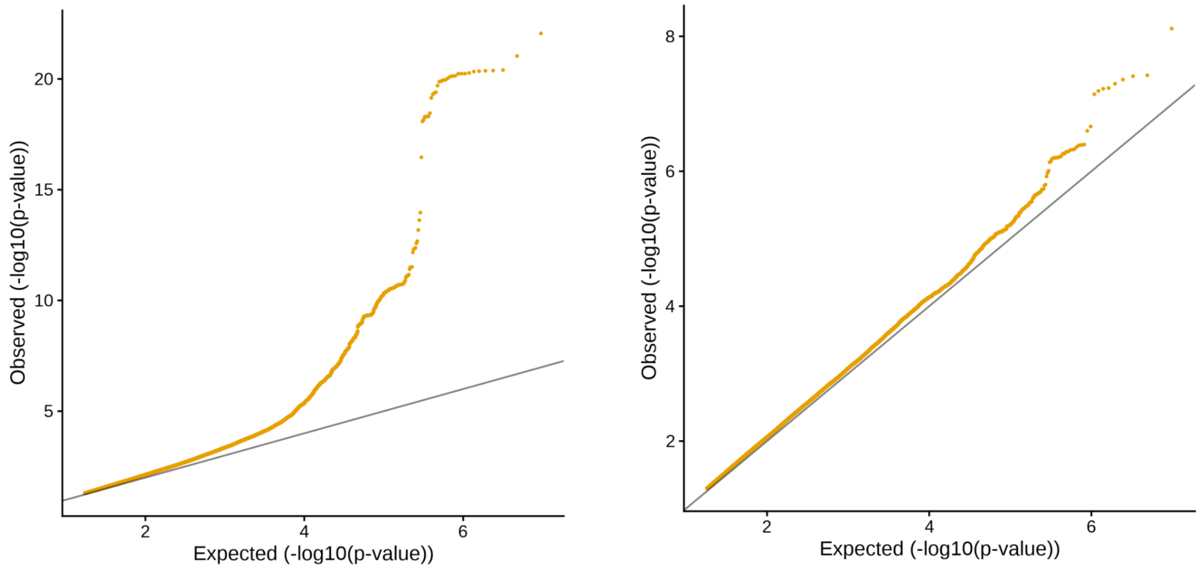

Supplementary Fig. 7. Quantile-quantile plot of preterm (n cases = 18,797, n controls = 260,246) and post-term delivery (n = 131,279, n cases = 15,972) GWAS meta-analysis. Observed p-values were two-sided, and were estimated using fixed effect inverse-variance weighted meta-analysis.

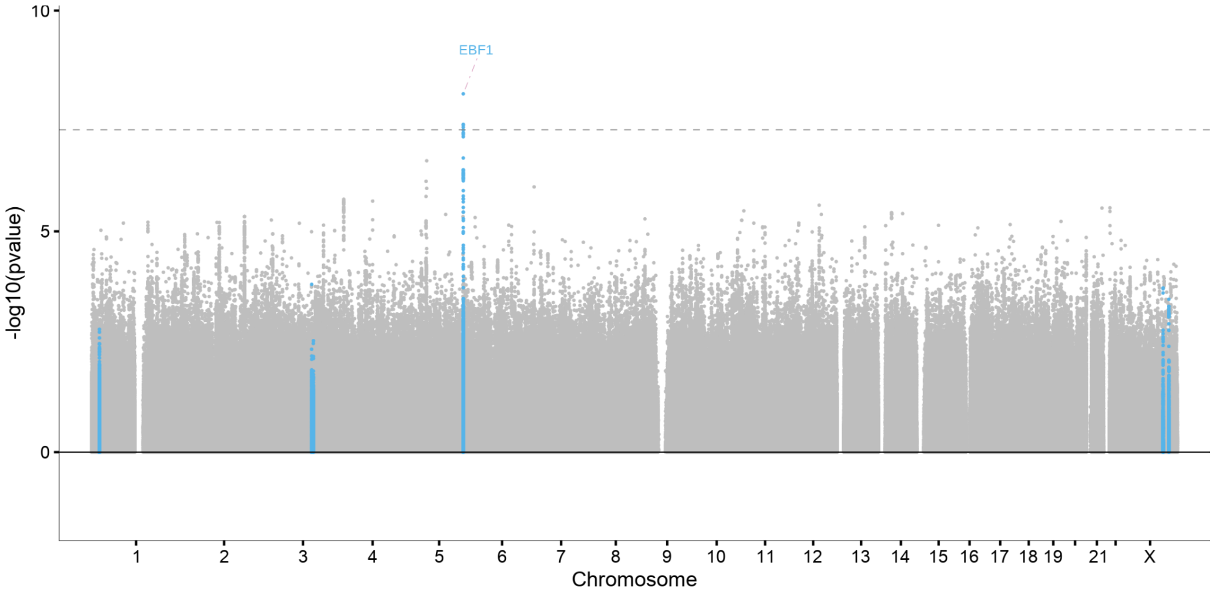

Supplementary Fig. 8. Manhattan plot of the meta-analysis of GWAS of post-term delivery (n = 131,279, n cases = 15,972). The x-axis shows the chromosome position and the y-axis the two-sided p-value of the fixed effect inverse-variance weighted meta-analysis. The dashed line represents the genome-wide significance threshold (p-value = 5×10-8). Each genome-wide significant locus is labeled by their nearest protein-coding gene.

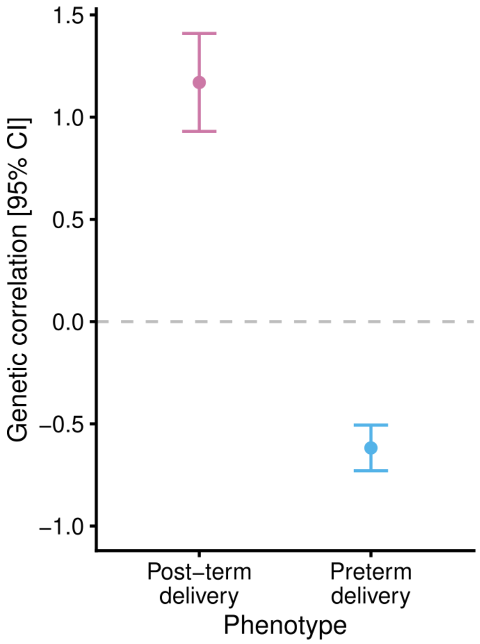

Supplementary Fig. 9. Genetic correlations between gestational duration (n = 195,555) and preterm (n cases = 18,797, n controls = 260,246) and post-term (n cases = 15,972, n controls = 115,307) deliveries. LD-score regression was used to estimate the genetic correlation with pre-computed LD-scores derived from samples with recent European ancestry from the 1000 Genomes Project. Error bars reflect 95% CI.

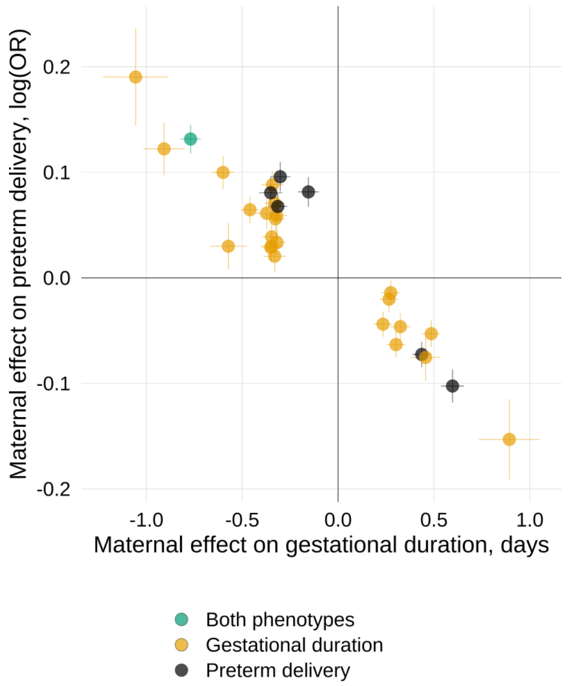

Supplementary Fig. 10. Maternal effects on gestational duration and preterm delivery for the index SNPs on the two phenotypes. The x-axis shows the effect of each SNP on gestational duration (days, n = 195,555), and the y-axis the effect of each SNP on preterm delivery (log(OR), n cases = 18,797, n controls = 260,246). Horizontal and vertical bars represent the standard errors.

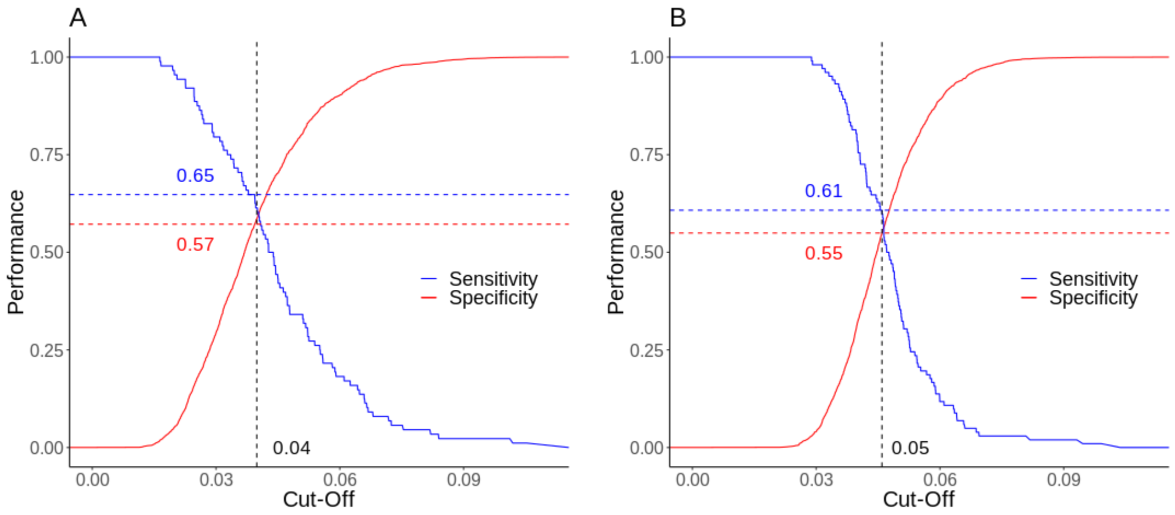

Supplementary Fig. 11. Polygenic score optimal sensitivity and specificity for preterm delivery (n = 3,943). Optimal probability threshold cut-off that maximizes sensitivity and specificity of the gestational duration (A) and preterm delivery (B) polygenic scores predicting preterm delivery.

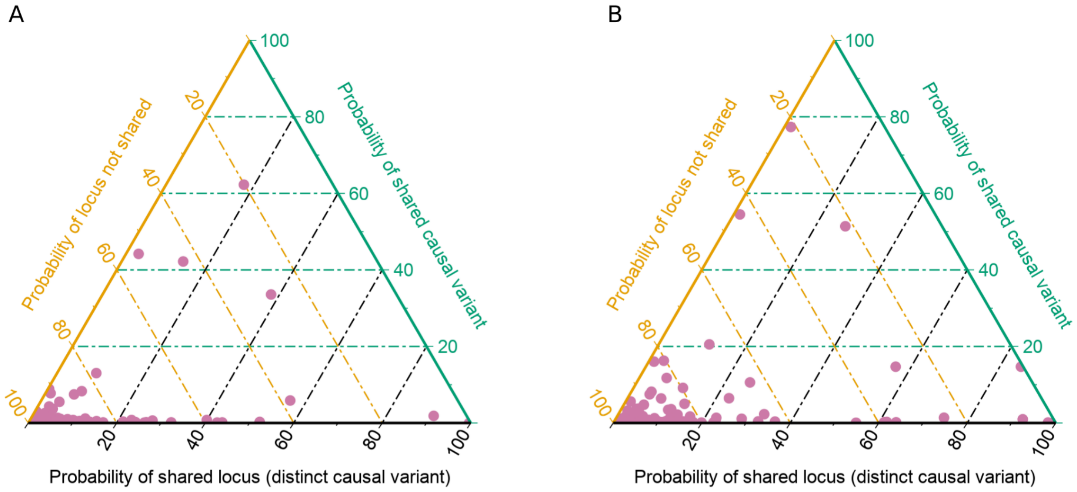

Testosterone (women)

Sex-hormone binding globulin

Supplementary Fig. 12. Ternary plot for the genome-wide colocalization probabilities of gestational duration and testosterone and sex-hormone binding globulin in women. The sum of all probabilities for each approximately independent region (n regions = 1,703) is 1, with each pink dot representing one of such regions. Lines are coloured according to the axis they belong to. All points in a horizontal green line have the same probability of “sharing the causal variant” between gestational duration and the hormone, points on a yellow line parallel to the right side of the triangle have the same probability of a “not sharing the locus” (i.e., locus has no association with either or both traits), and black lines parallel to the left side of the triangle have the same probability of a “sharing the locus, but with distinct causal variant”. Probabilities were obtained using coloc on previously published summary statistics for testosterone and sex-hormone binding globulin.

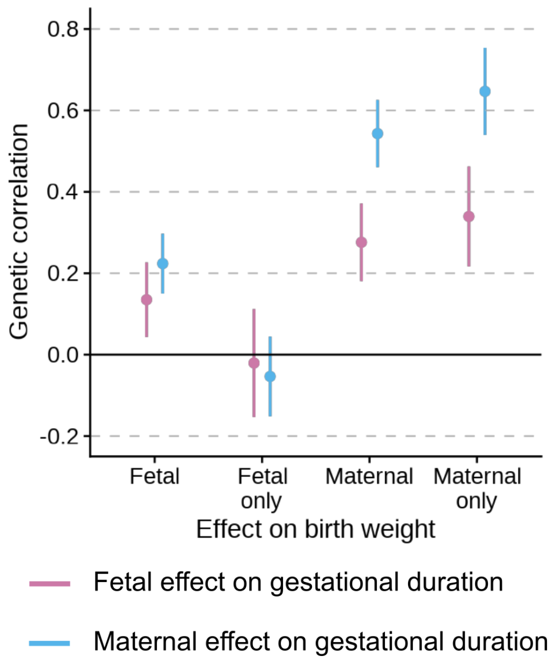

Supplementary Fig. 13. Genetic correlations (95% CI) between the maternal (blue, n = 195,555) and fetal (pink, n = 84,689) effects on gestational duration and the maternal (n = 210,248) and fetal (n = 297,356) effects on birth weight. The maternal only and fetal only effects on birth weight refer to the effects of the maternal genome after adjusting for the fetal effects and the effect of the fetal genome after adjusting for the maternal effects, respectively. Dots represent the genetic correlation estimate, and error bars the 95% CI.

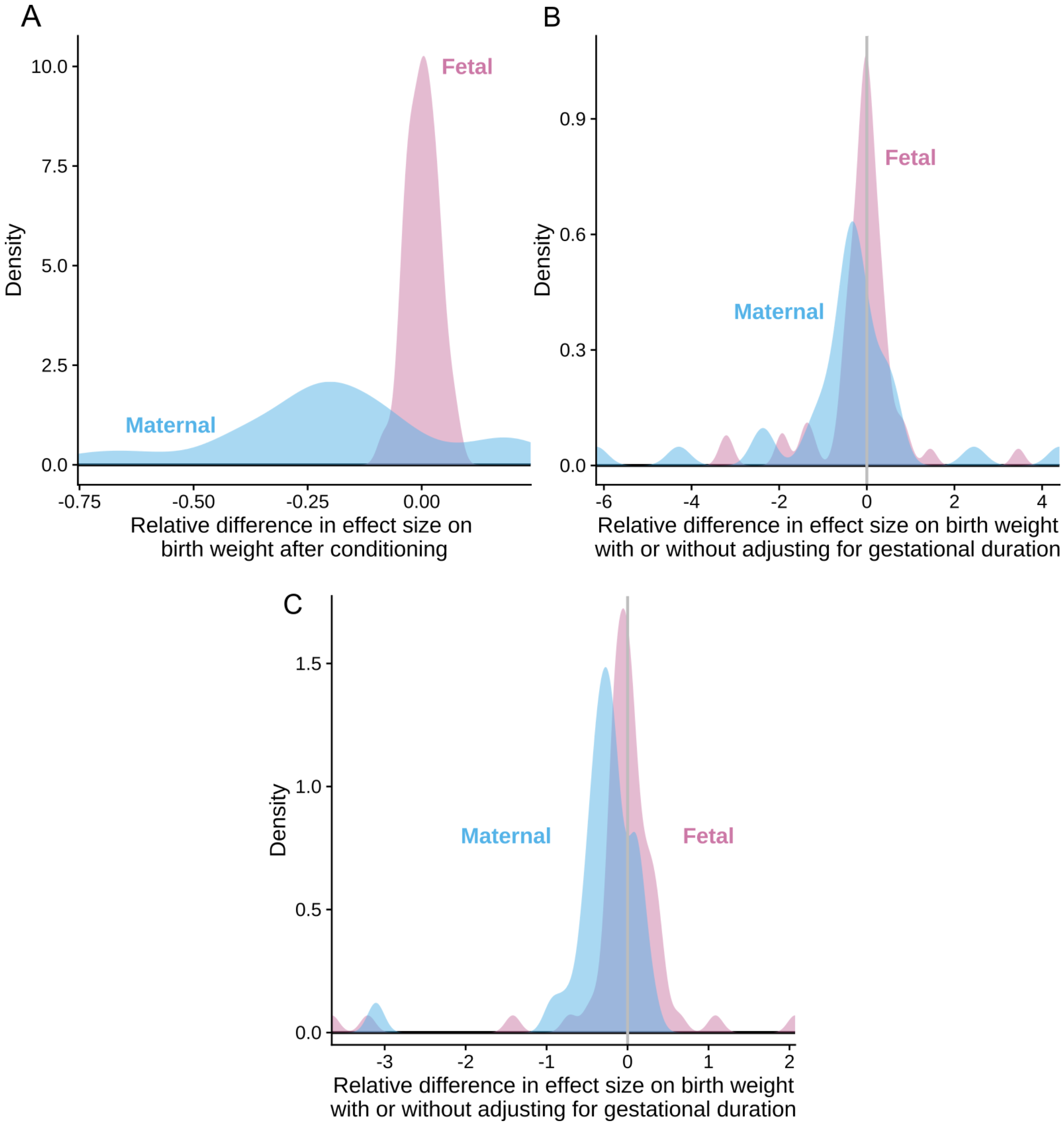

Supplementary Fig. 14. Relative difference in effect size on birth weight before and after adjusting by gestational duration. All analyses were performed on genetic variants classified as having a “Maternal Only” (n= 32) or a “Fetal Only” (n = 86) effect on birth weight in Warrington, et al, 2019, Nature Genetics [9]. In blue, relative difference in effect sizes for the maternal only effects on birth weight before and after conditioning; in pink, relative difference in effect sizes for the fetal only effects on birth weight after conditioning. A, Conditional analysis was performed using approximate multi-trait conditional and joint analysis in summary statistics. B, Relative difference in effect size on birth weight with and without adjusting for gestational duration using the maternal non-transmitted and paternal transmitted alleles (n = 21,060 parent-offsprings from MoBa) for the maternal and fetal effects on birth weight, respectively. C, Relative difference in effect size on birth weight with and without adjusting for gestational duration using genotype dosage in 32,511 mothers and 16,387 fetuses from Iceland.

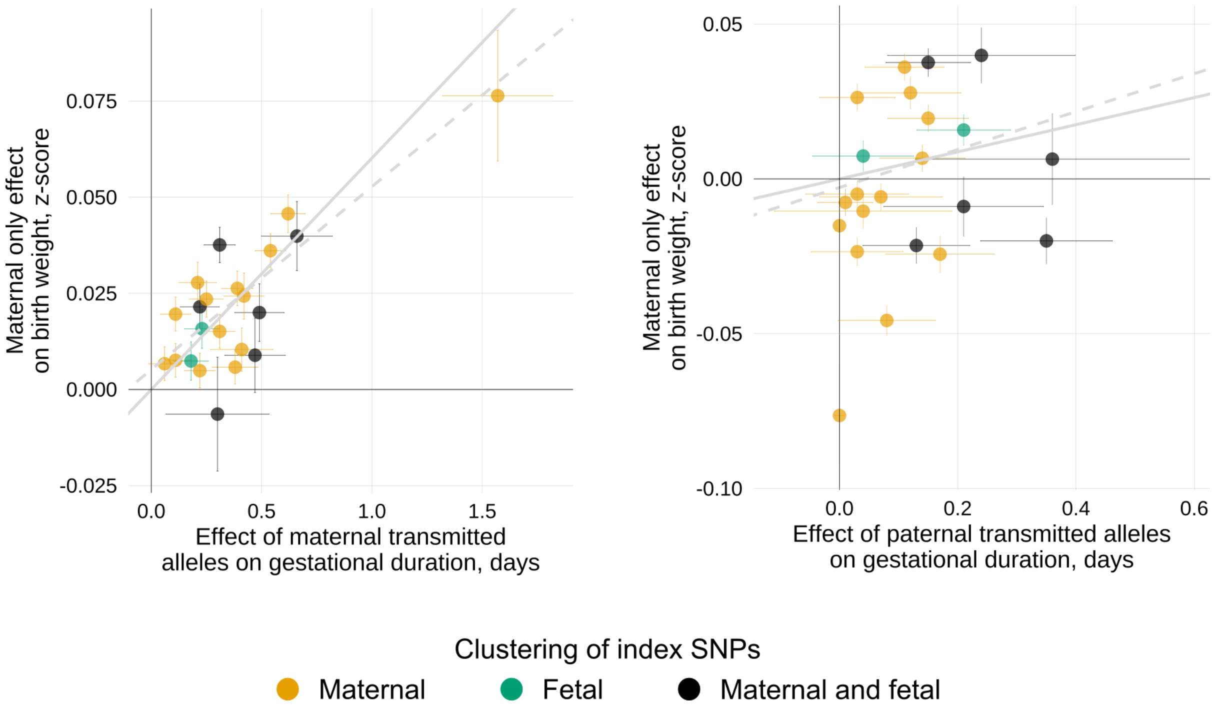

Supplementary Fig. 15. Association between maternal and paternal transmitted gestational duration-increasing alleles and maternal effect on birth weight. Scatterplot for two-sample Mendelian randomization analysis for the effect of gestational duration on birth weight (maternal effect). Each dot represents one of the gestational duration index SNPs. The x-axis shows the SNP effect of the maternal or paternal transmitted alleles on gestational duration in days (meta-analysis of multiple data sets, including data from Iceland, MoBa, HUNT, ALSPAC, DNBC, FIN, GPN, and HAPO; n = 136,833), and the y-axis the maternal only effect on birth weight in z-scores (weights were obtained from a previously published GWAS; n = 210,248). Horizontal and vertical error bars represent standard errors. The solid line depicts the inverse-variance weighted method estimate, and the dashed line the MR-Egger estimate. Colors represent the clustering of the index SNP.

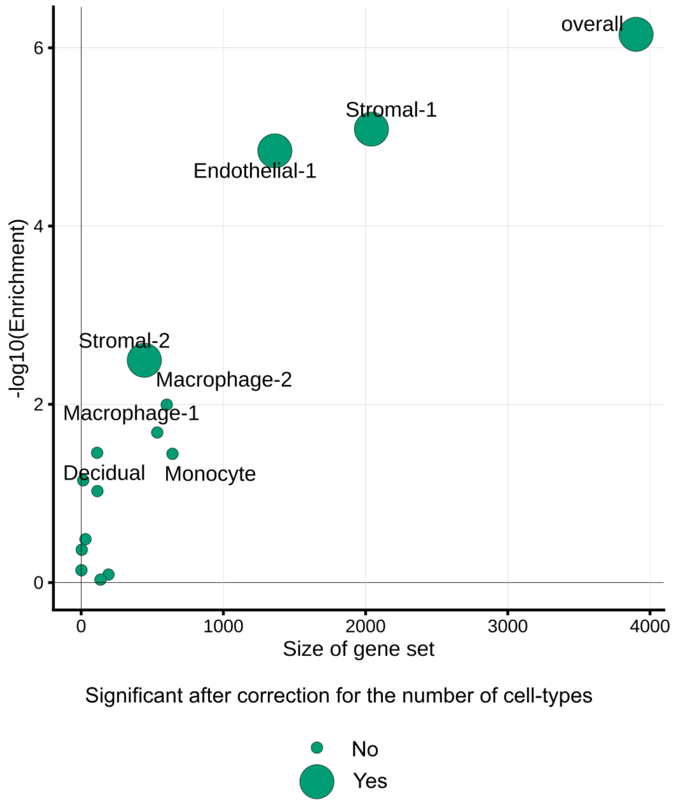

Supplementary Fig. 16. Gene set size and SNP-heritability enrichment of gestational duration for genes differentially expressed during labor in different cell types of the myometrium and overall. LD-score regression was used to partition heritability, and estimate the heritability enrichment p-value (two-sided) for each cell type and overall. The x-axis shows the number of genes included in each cell-type and overall; the y-axis shows the -log10(p-value) for enrichment. We calculated LD scores (European individuals from phase 3 of the 1000 Genomes project) for each set of genes differentially expressed at labor (± 100 kb), separately for each cell type and for the overall set of genes differentially expressed in the myometrium. Each dot represents a cell type, with larger showing significant heritability enrichment after correcting for the number of cell types. No adjustments for multiple comparisons were made.
