# Supplementary material for: Genetic effects on the timing of parturition and links to fetal birth weight
Source: Nat Genet. 2023 Apr 3;55(4):559–67. doi: 10.1038/s41588-023-01343-9 (PMC10101852; doi:10.1038/s41588-023-01343-9)
Supplement: Supplementary file 2 — Reporting Summary [file 41588_2023_1343_MOESM2_ESM.pdf]

nature portfolio

Corresponding author(s): Pol Sole-Navais and Bo Jacobsson

Last updated by author(s): Nov 14, 2022

Reporting Summary

Nature Portfolio wishes to improve the reproducibility of the work that we publish. This form provides structure for consistency and transparency in reporting. For further information on Nature Portfolio policies, see our Editorial Policies and the Editorial Policy Checklist.

Statistics

For all statistical analyses, confirm that the following items are present in the figure legend, table legend, main text, or Methods section.

n/a	Confirmed	
☐	☒	The exact sample size (*n*) for each experimental group/condition, given as a discrete number and unit of measurement
☐	☒	A statement on whether measurements were taken from distinct samples or whether the same sample was measured repeatedly
☐	☒	The statistical test(s) used AND whether they are one- or two-sided<br>*Only common tests should be described solely by name; describe more complex techniques in the Methods section.*
☐	☒	A description of all covariates tested
☐	☒	A description of any assumptions or corrections, such as tests of normality and adjustment for multiple comparisons
☐	☒	A full description of the statistical parameters including central tendency (e.g. means) or other basic estimates (e.g. regression coefficient) AND variation (e.g. standard deviation) or associated estimates of uncertainty (e.g. confidence intervals)
☐	☒	For null hypothesis testing, the test statistic (e.g. *F*, *t*, *r*) with confidence intervals, effect sizes, degrees of freedom and *P* value noted<br>*Give P values as exact values whenever suitable.*
☐	☒	For Bayesian analysis, information on the choice of priors and Markov chain Monte Carlo settings
☐	☒	For hierarchical and complex designs, identification of the appropriate level for tests and full reporting of outcomes
☐	☒	Estimates of effect sizes (e.g. Cohen's *d*, Pearson's *r*), indicating how they were calculated

Our web collection on statistics for biologists contains articles on many of the points above.

Software and code

Policy information about availability of computer code

Data collection	No software was used for data collection.
Data analysis	Code for the meta-analyis and down-stream analyses can be found at https://github.com/PerinatalLab/metaGWAS.<br>The following software were used for data analysis: R version 4.1.1 (https://www.r-project.org) with packages: data.table 1.14.0, dplyr 1.0.7, tidyr 1.1.3, scales 1.1.1, knitr 1.33, cowplot 1.1.0, ggrepel 0.9.1, showtext 0.9_3, tidyverse 1.3.1, fmsb 0.7.1, ggtern 3.3.5, MendelianRandomization 0.5.1, gridextra 2.3, dendextend 1.15.1, plyr 1.8.6, ggtree 3.0.1, kableextra 1.3.4, metafor 4.3.0; coloc v3.0; Python version 3.7.9 with packages: pandas 1.1.4, numpy 1.19.5, urllib3 1.26.6, scipy 1.5.3; PLINK v1.90b6.6 64-bit (https://www.cog-genomics.org/plink/); PLINK v2.00a2.3LM (https://www.cog-genomics.org/plink/2.0/); METAL - version released on 2011-03-25 (https://genome.sph.umich.edu/wiki/METAL); bcftools 1.9 (https://samtools.github.io/bcftools/bcftools.html); ensembl-vep 106.1 (https://grch37.ensembl.org/Homo_sapiens/Tools/VEP); qctool v2.0.8 (https://www.well.ox.ac.uk/~gav/qctool/); GCTA 1.93.2beta (https://yanglab.westlake.edu.cn/software/gcta/#Overview); LCV (https://github.com/lukejoconnor/LCV, cloned the 24/08/2021); Python 2.7 with the following packages: scipy 0.18, pandas 0.20, numpy 1.16; LDSC v1.0.1 (https://github.com/bulik/ldsc, cloned the 27/05/2020); bedtools v2.29.2 (https://bedtools.readthedocs.io/en/latest/); BOLT-LMM v2.3 (https://alkesgroup.broadinstitute.org/BOLT-LMM/BOLT-LMM_manual.html#x1-5700012); LDpred2 (https://privefl.github.io/bigsnpr/articles/LDpred2.html); SHAPEIT2 (https://mathgen.stats.ox.ac.uk/genetics_software/shapeit/shapeit.html); EAGLE v2.3 (https://alkesgroup.broadinstitute.org/Eagle/#x1-40002.1)

For manuscripts utilizing custom algorithms or software that are central to the research but not yet described in published literature, software must be made available to editors and reviewers. We strongly encourage code deposition in a community repository (e.g. GitHub). See the Nature Portfolio guidelines for submitting code & software for further information.

Data

Policy information about availability of data

All manuscripts must include a data availability statement. This statement should provide the following information, where applicable:

- Accession codes, unique identifiers, or web links for publicly available datasets
- A description of any restrictions on data availability
- For clinical datasets or third party data, please ensure that the statement adheres to our policy

Cohorts should be contacted individually for access to raw genotype data, as each cohort has different data access policies. Summary statistics from the meta-analysis excluding 23andMe for each phenotype are available at the EGG website (egg-consortium.org/) and access to the weights for constructing the polygenic score of gestational duration excluding 23andMe are available at the PGS Catalog (https://www.pgscatalog.org/, score ID: PGS002806). Access to the full set, including 23andMe results, can be obtained after approval from 23andMe is presented to the corresponding author or by completion of a Data Transfer Agreement (https://research.23andme.com/dataset-access/), which exists to protect the privacy of 23andMe participants. Access to the Danish National Birth Cohort (phs000103.v1.p1), Hyperglycemia and Adverse Pregnancy Outcome (phs000096.v4.p1), and Genomic and Proteomic Network (phs000714.v1.p1) individual-level phenotype and genetic data can be obtained through dbGaP Authorized Access portal (https://dbgap.ncbi.nlm.nih.gov/dbgap/aa/wga.cgi?page=login). The informed consent under which the data or samples were collected is the basis for determining the appropriateness of sharing data through unrestricted-access databases or NIH-designated controlled-access data repositories. The summary statistics used in this publication other than the one generated are available at the following links: fetal GWAS of gestational duration (http://egg-consortium.org/gestational-duration-2019.html), fetal and maternal GWAS of birth weight (http://egg-consortium.org/birth-weight-2019.html), miscarriage (http://www.geenivaramu.ee/tools/misc_sumstats.zip), age at first birth, oestradiol (women), endometriosis, number of live births and age at menarche (http://www.nealelab.is), age at menopause (https://www.reprogen.org), testosterone (women) 59, SHBG, testosterone and CBAT (https://doi.org/10.6084/m9.figshare.c.5304500.v1), pelvic organ prolapse and leiomyoma of the uterus (https://www.finngen.fi/fi), polycystic ovary syndrome (https://www.repository.cam.ac.uk/handle/1810/283491 and https://www.finngen.fi/fi) and pre-eclampsia (European Genome-phenome Archive, https://ega-archive.org, EGAD00010001984). Pan-UK Biobank data is available at https://pan.ukbb.broadinstitute.org/. For pre-computed LD scores for European populations (https://data.broadinstitute.org/alkesgroup/LDSCORE/eur_w_ld_chr.tar.bz2), and for multi-tissue gene expression pre-computed stratified LD scores (https://alkesgroup.broadinstitute.org/LDSCORE/LDSC_SEG_ldscores/Multi_tissue_gene_expr_1000Gv3_ldscores.tgz). eQTL data from GTEx is available at https://gtexportal.org/home/ and from endometrium at http://reproductivegenomics.com.au/shiny/endo_eqtl_rna/. Protein QTL data was obtained from https://www.omicscience.org/apps/pgwas/. Genome Reference Consortium Human Build 37 (hg19) available at https://www.ncbi.nlm.nih.gov/data-hub/genome/GCF_000001405.13/.

Human research participants

Policy information about studies involving human research participants and Sex and Gender in Research.

Reporting on sex and gender

Findings from this work largely apply to one sex (women). The main GWAS meta-analysis was performed using only genotype and pregnancy information from women. In the analysis to distinguish maternal and fetal effects, we used parental data (including fathers, men) as well their offspring. Sex was determined using genotype data, and inconsistencies were excluded from the analysis.
One section includes the use of a sex-stratified GWAS of adult horomone levels, due to large differences in the genetic effects on such hormones between sexes.

Population characteristics

The study included pregnant women of recent European ancestry with a singleton live-birth. Over 70% of the samples were recruited in Nordic countries (Iceland, Norway, Finland and Denmark), where the prevalence of spontaneous preterm delivery is 3%. Overall, the median preterm delivery rate was 9.2%, including case-control studies ascertained for preterm delivery. Post-term delivery rate was notably higher (approx. 13.1%) considering that no case-control studies were included. Median maternal age at birth ranged between 20-30 years old (median = 25), and nulliparous women (first pregnancy with delivery after 22 gestational weeks) were approximately half the sample size.

Recruitment

Recruitment and data collection was performed independently by each participating cohort, and is detailed in the Supplementary Note.
Recruitment in cohorts participating in this GWAS meta-analysis can be largely grouped in three distinct categories: population-based, hospital-based or direct-to-consumer. Several cohorts were case-controls for preterm delivery, and have only been included as part of the preterm delivery GWAS meta-analysis. Most population-based cohorts originate from Nordic countries, where genetic data was linked to medical birth registers from each country. Only data from one direct-to-consumer genotyping company was employed (23andMe). Hospital-based cohorts were the most common, but overall accounted for a relatively small sample size. Participation bias is potentially the major affecting our analysis, but recent studies suggest it is difficult to determine how it may impact genotype-phenotype associations, particularly in meta-analyses. While molecular/ physical traits are less likely to being affected by participation bias, recall in last date of menstrual period may have biased the estimates of gestational duration. Samples included in this study have been largely recruited in Nordic countries, where rates of preterm delivery are amongst the lowest in the world.

Ethics oversight

All participants provided a signed written informed consent and study protocols were approved by each respective Institutional review boards. Ethic statements from each participating cohort are detailed in the Supplementary Note. Briefly, approval of study protocols was obtained from Ethical and Independent Review Services (https://eandireview.com/), Ethics Committee and the Local Research Ethics Committees (Bristol and Weston, Southmead, and Frenchay Health Authorities), Bradford Local NHS Research Ethics Committee, South East Multi-centre Research Ethics Committee and the Joint UCL/UCLH Committees on the Ethics of Human Research (Committee A), Institutional Review Board of the Children's Hospital of Philadelphia, the reproductive health protocol, the National Bioethics Committee (Iceland) following evaluation of the Icelandic Data Protection Authority, Regional Scientific Ethical Committee of the Region of Mid Jutland and the Danish Data Protection Agency, the Regional Scientific Ethical Committee of Copenhagen, the Ethics Review Committee of the University of Tartu, the North and East Devon (UK) Local Research Ethics Committee, the Ethics Committee of the Helsinki University

Central Hospital, the Investigational Review Boards at all participating institutions of GPN, The Regional Committee for Medical Research Ethics, the Regional Committee for Medical Research Ethics, Southern Norway, Oslo, Norway, the CHUS ethic committee board, the Regional Ethics Committee for Medical Research Ethics South East Norway and the Women's and Newborn Health Service.

Note that full information on the approval of the study protocol must also be provided in the manuscript.

Field-specific reporting

Please select the one below that is the best fit for your research. If you are not sure, read the appropriate sections before making your selection.

☒ Life sciences ☐ Behavioural & social sciences ☐ Ecological, evolutionary & environmental sciences

For a reference copy of the document with all sections, see nature.com/documents/nr-reporting-summary-flat.pdf

Life sciences study design

All studies must disclose on these points even when the disclosure is negative.

Sample size	The research sample consisted of 195,555 samples for gestational duration, 276,218 samples for preterm delivery (18,797 cases) and 115,307 samples for preterm delivery (15,972 cases) from 18 different cohorts. We included previously published data from 23andMe (Zhang et al., 2017) as well as data from different cohorts from the Early Growth Genetics Consortium, the Estonian Biobank, the Danish Blood Donor Study Genomic Consortium, the Norwegian Mother, Father and Child cohort study, The Trøndelag Health Study and deCODE genetics (Iceland). To study the effects of the parental transmitted and non-transmitted alleles, we pooled together results from the largest parent-offspring datasets available to date (n = 136,833 parent-offspring trios or mother-child duos). These samples were partly included in the discovery analysis (main GWAS meta-analysis). Other data generated in this study are included in the main article or in the Supplementary Tables or Figures.<br>For analyses where Mendelian randomization was performed, we used to distinct, complementary methods: two- and one-sample Mendelian randomization. The former was chosen to use the largest sample size as possible, given that prior studies on the relationship between sex-hormones and gestational duration were lacking (limiting the calculation of study sample size). One-sample Mendelian randomization was mainly carried out by deriving polygenic scores from lead SNPs (genome-wide significant) for a number of traits (sex hormones and birth weight). This analysis was carried out primarily to detect whether the effect observed using two-sample Mendelian randomization was driven by the maternal or the fetal genome, and was mainly used as an exploratory analysis. With regards of the effects of fetal growth (fetal effects on birh weight) on gestational duration, previous evidence suggested an effect of 3 days per standard deviation in birth weight (n = 10,000). In our analysis, we pooled data in >32,000 parent-offsprings, which we consider was sufficient for a reduction in effect size of 50%. As a general note, power calculations for the effects of parental transmitted and non-transmitted alleles is complex for several reasons: previous evidence is difficult to gather, observed effects using phenotypic associations may be misleading (i.e., in cases of opposite effect direction between maternal and fetal genomes) and additive effects are assumed.
Data exclusions	Data exclusions were performed on a cohort-specific basis. Whenever the information was available, medically-initiated labors were excluded. We excluded pregnancies lasting <140 days (20 completed weeks) or >310 days (44 completed weeks), multiplets as well as women with health complications prior to or during pregnancy and congenital fetal malformations.
Replication	No direct replication were performed of the main results, given that we pooled data from most cohorts with gestational duration (or preterm delivery) and genotype data available. We replicated the previously reported gestational duration loci (Zhang et al., 2017) in an out-of-sample analysis.
Randomization	Not applicable - observational study.
Blinding	Not applicable - observational study.

Reporting for specific materials, systems and methods

We require information from authors about some types of materials, experimental systems and methods used in many studies. Here, indicate whether each material, system or method listed is relevant to your study. If you are not sure if a list item applies to your research, read the appropriate section before selecting a response.

Materials & experimental systems

n/a	Involved in the study
☒	☐ Antibodies
☒	☐ Eukaryotic cell lines
☒	☐ Palaeontology and archaeology
☒	☐ Animals and other organisms
☒	☐ Clinical data
☒	☐ Dual use research of concern

Methods

n/a	Involved in the study
☒	☐ ChIP-seq
☒	☐ Flow cytometry
☒	☐ MRI-based neuroimaging
